# IL-15 Participates in the Pathogenesis of Polycystic Ovary Syndrome by Affecting the Activity of Granulosa Cells

**DOI:** 10.3389/fendo.2022.787876

**Published:** 2022-02-18

**Authors:** Yan Liu, Zhi Li, Yang Wang, Qingqing Cai, Haiou Liu, Congjian Xu, Feifei Zhang

**Affiliations:** ^1^ Obstetrics and Gynecology Hospital, Fudan University, Shanghai, China; ^2^ Shanghai Key Laboratory of Female Reproductive Endocrine Related Diseases, Shanghai, China; ^3^ Department of Obstetrics and Gynecology, Shanghai Medical School, Fudan University, Shanghai, China

**Keywords:** PCOS, chronic inflammation state, IL-15, KGN, mouse primary granulosa cells

## Abstract

**Background:**

Low-grade chronic inflammation may contribute to the pathogenesis of polycystic ovary syndrome (PCOS). Interleukin-15 (IL-15) is a proinflammatory cytokine involved in the development of chronic inflammation leading to obesity-associated metabolic syndrome. However, the concentration of IL-15 in follicular fluid of patients with PCOS has yet been evaluated.

**Objectives:**

The aim of this study is to evaluate the expression level of IL-15 in both patients with PCOS and PCOS mice model and investigate the functional effect of IL-15 on ovarian granulosa cells.

**Methods:**

The level of IL-15 in follicular fluid (FF) was measured using cytokine array and enzyme linked immunosorbent assay (ELISA) in two cohorts from 23 PCOS patients and 18 normo-ovulatory controls. PCOS mice model was induced by subcutaneously implanted with letrozole pellet for 21 days. The expression level of IL-15 in serum, ovarian, and subcutaneous adipose tissue in PCOS mice model was measured by ELISA, real-time polymerase chain reaction (RT-PCR), immunohistochemistry (IHC), and immunofluorescence. The effect of IL-15 on the proliferation and apoptosis of the KGN cells and mouse ovarian granulosa cells (GCs) were detected by CCK-8 assay and flow cytometry, respectively. Transcript expression of 17α-hydroxylase17,20-lyase (*CYP17A1*), cytochrome P450 family 19 subfamily A member 1(*CYP19A1*), FSH receptor (*FSHR*), steroidogenic acute regulatory protein (*StAR*), and proinflammatory cytokine were quantified using RT-PCR. The protein level and phosphorylation level of p38 MAPK and JNK are detected by Western blot. Concentration of dehydroepiandrosterone sulfate (DHEAS) and progesterone (P)were measured by ELISA.

**Results:**

IL-15 expression in follicular fluid of patients with PCOS was significantly elevated compared with the control group, and similar results were observed in the ovarian and subcutaneous adipose tissue of PCOS mice models. Furthermore, the elevated FF IL-15 levels have a positive correlation with the serum testosterone levels. FSHR co-localized with IL-15 indicating that IL-15 production originate from ovarian granulose cells. IL-15 treatment inhibited proliferation and promoted apoptosis of KGN cells and mouse GCs. Moreover, IL-15 upregulated the transcription levels of *CYP17A1*, *IL-1b* and *Ifng* KGN cells. Similar results were observed in mouse GCs except concentration of DHEAS was higher in IL-15 treatment. IL-15 promoted p38 MAPK and JNK phosphorylation in KGN cells, treating KGN cells with p38 MAPK inhibitor SP600125 and JNK inhibitor SB203580 could reverse the effect of IL-15 on the proliferation and function of KGN cells.

**Conclusion:**

The results indicate that IL-15 is involved in the pathogenesis of PCOS potentially by affecting survival, the inflammation state and steroidogenesis of granulosa cells. The practical significance of this association between IL-15 and the pathogenesis of PCOS needs further investigation.

## Introduction

Polycystic ovary syndrome (PCOS), characterized with hyperandrogenism and ovulatory dysfunction, irregular menstruation, and insulin resistance, is one of the most common endocrine and metabolic disorders in reproductive-age women (1). PCOS is the main cause of infertility in women of reproductive age. The global prevalence of PCOS ranges from 4% to 20% owing to differences in diagnostic criteria and population assessed in different geographic areas (2, 3). Due to its obscure etiology and high heterogeneity of clinical manifestations, the available therapeutic strategies for PCOS mainly rely on symptoms while there is no cure yet. The complex interaction between genetical and environmental factors, metabolic alterations, neuroendocrine and immune systems is supposed to play a role to the pathogenesis of PCOS (4–6). Moreover, physiological inflammation occurs in female reproductive tract during ovulation, menstruation, implantation, and labor at term, the establishment of low-grade chronic inflammation may participate in PCOS etiology (7, 8). PCOS patients have permanently elevated serum and ovarian levels of inflammatory markers interleukin-2 (IL-2), IL-6, IL-18, interferon-γ (IFN-γ), and tumor necrosis factor-α (TNF-α) compared with normal controls (9–11). The association between inflammatory cytokines and ovarian dysfunction implies that inflammation might be reckoned as the most potent risk factor of PCOS (12). Further investigating the role of inflammatory mediators in the commencement and development of PCOS could be critical for better understanding the pathophysiology of the disease and developing a potential therapeutic target.

IL-15 is secreted by many cell types, including both immune and nonimmune cells such as T-lymphocytes, macrophages, neutrophils and skeletal muscle cells (13, 14). IL-15 has attracted considerable attention for its beneficial effects, including improving lipid and glucose metabolism, suppressing white adipose tissue inflammation, enhancing mitochondrial function, and attenuating endoplasmic reticulum stress (15, 16). In contrast with beneficial effects of IL-15, IL-15 was also reported to participate in chronic inflammation of adipose tissue leading to obesity-associated metabolic syndrome, which absent in IL-15 KO mice prevented accumulation of fat in the white adipose tissue and promoted lipid utilization *via* adaptive thermogenesis (17). Furthermore, serum IL-15 concentrations were higher in overweight subjects, suggesting that adipose tissue depots might be a source of IL-15 (18). IL-15 was increased in follicular fluid (FF) from women with endometriosis, suggesting that IL-15 may have impaired oocyte quality leading to lower fertilization rates (19). IL-15 concentration in FF of follicles with immature oocytes were significantly higher than those with mature oocytes, suggesting that IL-15 should be investigated as a possible predictive factor for oocyte maturity (20). Adverse correlation between FF IL-15 concentration and maturity of oocyte, therefore, we aimed to investigate the pathogenesis role of IL-15 in women with PCOS.

Granulosa cells (GCs) are the predominant somatic cell type of the ovarian follicle and involved in folliculogenesis through proliferation, acquisition of gonadotropic responsiveness, steroidogenesis and production of autocrine/paracrine factors (21). Increased apoptosis of GCs has been proved in patients with PCOS and PCOS animals, although the underlying mechanisms of apoptosis in GCs have not been fully revealed (22–24). Hyperandrogenism directly induces apoptosis of GCs by stimulating an intrinsic pathway and decreasing the production of follicular growth factors (25–28). Immunosuppressive cytokines such as TGF-β also induced GCs apoptosis during the follicular development in PCOS rats (29). Although the IL-15 concentration in FF is negatively related to the maturity of oocytes, the effect of IL-15 on the proliferation and apoptosis of GCs remains unknown. The MAPK signaling pathway is one of the important pathways of IL-15. The activation of this signaling pathway affects the proliferation and apoptosis of target cells and the release of inflammatory factors (30, 31). And interestingly, phosphorylation of the MAPK pathway is involved in the expression of androgen synthesis related enzymes StAR and CAP17A1 (32, 33). We hypothesize that IL-15 will affect the proliferation of granulosa cells and the expression of genes related to androgen synthesis through the above signaling.

Knowledge in the association between FF IL-15 concentration and testosterone in women with PCOS could provide new insight into the pathogenesis of PCOS. this study aimed to detect the FF IL-15 concentration in women with PCOS, examine the association of FF IL-15 concentration with serum testosterone and explore the effect of IL-15 on the biological activities of GCs.

## Materials and Methods

### Study Subjects and Sample Collection

This study was approved by the Ethics Committee of Obstetrics and Gynecology Hospital affiliated Fudan University. Informed written consent was obtained from all the participants. Eligible women who had undergone IVF were recruited from September 2020 to January 2021. PCOS was diagnosed based on the Rotterdam criteria with two of the following: oligo and/or anovulation, polycystic ovarian morphology, and clinical and/or biochemical signs of hyperandrogenism (in this study, hyperandrogenism was defined as T>51ng/dl、the menstrual cycle exceeds 45 days and the number of small follicles visible on both sides of the ovary under ultrasound is ≥12). The control group included women who seek treatment for tubal infertility or male factors, with normal ovarian reserve (regular menstrual cycles, and normal ovarian morphology) who has normal BMI, the menstrual cycle is 28-35 days and the number of small follicles in a unilateral ovary under ultrasound <10. Women with endometriosis, cancer, or other medical disorders that could affect folliculogenesis were excluded.

FF samples were collected from 3 PCOS patients and 3 controls of the participants for cytokine array analysis. Patients included met all the three items of Rotterdam criteria. The clinical, hormonal characteristics were compared between the two subgroups (**Table 1**). FF samples from an additional 20 PCOS patients and 15 controls of the participants were collected for ELISA validation of the identified cytokines, and the clinical, hormonal characteristics were presented in **Table 2**. All subjects underwent controlled ovarian stimulation using the standard IVF antagonist stimulation regimen protocol. FF was collected by transvaginal ultrasound-guided aspiration, 36 h after the administration of recombinant human chorionic gonadotropin. Only clear FF samples with no macroscopic blood contamination were included. After oocyte isolation, the FF samples were centrifuged at 800 g for 10 min to remove pellets. The supernatant was then separated and stored at -80°C for future use.

**Table 1:** Clinical information of the participants for cytokine array analysis.

	PCOS (n = 3)	CON (n=3)	P	R
**Age (year)**	30.67 ± 6.03	32.00 ± 1.00	0.725	
**FSH (mIU/mL)**	7.23 ± 1.68	7.53 ± 0.31	0.782	
**LH (mIU/mL)**	10.23 ± 4.02	4.63 ± 1.70	0.093	
**E2 (pg/ml)**	45.33 ± 7.02	40.00 ± 8.72	0.456	
**T (ng/dl)**	56.00 ± 10.15	31.00 ± 2.64	0.015	0.8099
**LH/FSH**	2.69 ± 0.63	0.64 ± 0.33	0.02	0.8617
**Height (m)**	1.657 ± 0.111	1.61 ± 0.02	0.541	
**Weight (kg)**	68.33 ± 7.23	48.00 ± 4.58	0.015	0.8087
BMI* (kg/m2)	24.89 ± 1.18	18.42 ± 1.3	0.003	0.9106

**Table 2:** Clinical information of the participants for ELISA of IL-15.

	PCOS (n=20)	CON (n=15)	P	R
**Age (year)**	29.61 ± 4.43	32.50 ± 4.14	0.055	
**FSH (mIU/ml)**	6.75 ± 1.46	7.93 ± 2.31	0.075	
**LH (mIU/ml)**	7.53 ± 6.93	4.58 ± 1.64	0.118	
**E2 (pg/ml)**	39.25 ± 13.06	40.67 ± 11.05	0.744	
**T (ng/dl)**	57.80 ± 16.45	41.00 ± 8.18	0.001	0.2847
**LH/FSH**	1.04 ± 0.84	0.5937 ± 0.19	0.055	
**Height (m)**	1.61 ± 0.040	1.61 ± 0.05	0.965	
**Weight (kg)**	61.45 ± 8.43	60.77 ± 8.40	0.815	
BMI* (kg/m2)	23.84 ± 3.25	23.63 ± 3.79	0.861	

*For **Tables 1** and **2**: BMI (body mass index)=weight(kg)/height(m)².

### Cytokine and Chemokine Array

A cytokine and chemokine array (Proteome Profiler TM Human XL Cytokine Array Kit, R&D Systems, MN, USA) was used to detect the changes in 102 cytokines and chemokines in follicular fluid from PCOS and non-PCOS patients according to the manufacturer’s instructions. Briefly, samples were incubated on the membrane overnight at 4°C on a rocking shaker. The membranes were washed and incubated with a cocktail of biotinylated dectection antibody, and then the membrane was incubated with Strepatividin-HRP and chemiluminescent detection reagents. The chemiluminescent signal on each membrane was collected using an Amershan Imager 600 (GE Healthcare Life Sciences, Pittsburgh, PA, USA). The intensity (Pixel density) of each spot was quantified using HL Image++ (Western Vision Software, Salt Lake City, UT, USA), and corrected for background intensity and normalized to the membrane’s positive control.

### ELISA Validation

The differential abundance of IL-15 was validated by ELISA using FF samples of 20 patients with PCOS and 15 control participants. Concentrations of IL-15 in the FF samples were measured using the commercial human IL-15 ELSIA kits (Multi Science, Hangzhou, China) according to the manufacturer’s instructions.

### PCOS Modeling

Three-week-old female C57BL/6 mice (Jiesjie laboratory animal co. LTD, Shanghai, China) were maintained in a 12h light/12 h dark cycle with free access to rodent feed and water. All procedures were carried out followed the guidelines provided by the Fudan University Institutional Animal Ethical Committee. Mice were divided randomly into two subgroups: control was subcutaneously implanted with a placebo and PCOS was subcutaneously implanted with 3mg letrozole (LTZ) pellet (Innovative Research of American, Sarasota, FL, USA) for 21 days. At the end of experiment, all mice were euthanized by intraperitoneal injection with pentobarbital sodium. Ovary was dissected out and fixed by paraformaldehyde to paraffin embedding. The animal study was approved by the Ethics Committee of Fudan University.

### Vaginal Smears

Viginal smears of all mice were collected and for determination of estrous cycle, Giemsa staining was used, and stages of estrous cycle were determined microscopically.

### Glucose Tolerance Tests

Mice were fasted for 12 h before the glucose tolerance tests (GTT) and 4h before the insulin tolerance tests (ITT). Glucose levels were measured by tail vein blood sampling using Accu-Chek Performa blood glucose analyzer (Roche Diagnostics). The mice were intraperitoneally injected with D-glucose (2g/kg body weight) for GTT or insulin (1 IU/kg body weight) for ITT after measurement of fasting glucose levels, and tail samples were collected at 15,30,60,90 and 120 min after the IP injection for glucose level detection. And the level of fasting insulin was measured by ELSIA (Multi Science, Hangzhou, China). And HOMA-IR (homeostasis model assessment of insulin resistance) index was calculated refer to (34).

### Serum Analysis

Serum testosterone (T), dehydroepiandrosterone sulfate (DHEAS), luteinizing hormone (LH), follicle-stimulating hormone (FSH) concentrations were measured by corresponding ELISA kits (Sino-UK bio, Beijing, China). Moreover, serum and ovarian tissue homogenates IL-15 concentration was determined through a mice ELISA kit (Multi Science, Hangzhou, China).

### Hematoxylin and Eosin Staining

Hematoxylin and eosin (H&E) staining was performed for ovary following deparaffinization and rehydration. 5μm sections were stained using hematoxylin followed by eosin staining and subjected to graded alcohol dehydration. The histopathological analysis of the ovary was evaluated by two independent viewers (Yan Liu, Zhi Li) who were blinded to the group information.

### Immunohistochemistry and Immunofluorescence

Immunohistochemistry (IHC) staining of IL-15 was used an immunohistochemical SP kit (Origene, Rockville, MD, USA) following the manufacturer’s instructions. Briefly, tissue sections were boiled in antigen retrieval buffer. After cooling, sections were incubated with 3% hydrogen peroxide solution at room temperature for 15 min followed with 10% goat serum as an antigen-blocking buffer for 30 min at 37°C. Sections were then incubated with Rabbit antibody against IL-15 (1:100, Affinity Bioscience, OH,USA) overnight at 4°C in a humid chamber. HRP-conjugated secondary antibody was applied for 30 min and visualized with 3’3-diaminobenzideine (DAB). Observations were made using a Nikon Eclipse 80i (Nikon, Tokyo, Japan) microscope. The positive staining areas were measured by Image J software (NIH, USA). Immunofluorescence staining was applied by a multiple fluorescent staining kit (Absin Bioscience, Shanghai China) according to the manufacturer’s instructions. The sections were incubated with primary antibody against IL-15 (1:100), and FSHR (1:500, Service Bio, Wuhan, China). Images were captured using a laser confocal microscope (Leica TCS SP8, Germany).

### Cell Culture

KGN (human ovarian granulosa cell tumor) cells were obtained from Shandong University and were cultured with DMEM/F12 medium (Gibco, Grand Island, NY, USA) supplemented with 10% fetal bovine serum (Gibco) and 1% penicillin-streptomycin in a humidified incubator at 37°C with 5% CO2. Mouse primary granulosa cells (mGCs) were isolated from ovaries of 3 weeks age female C57 mice. Ovaries were dip on ice in Lebovitz’s-15 medium (Sigma-Aldrich, USA) with 10% fetal bovine serum and 1% penicillin-streptomycin. A 25-gauge needle was used to separate surrounding adipose and envelope tissues then puncture the ovary to release the GCs. The cell suspension was centrifuged at 100 rpm for 5 min, resuspended with McCoy’s 5A medium (Sigma Aldrich, USA) supplemented with 5% fetal bovine serum and 1% penicillin-streptomycin. To further investigate the involvement of MAPK signaling pathways in IL-15–induced apoptosis and CYP17A1 abundance, the cells were preincubated with or without p38 MAPK inhibitor SB203580 (10 uM, Selleck Chemicals, USA) (35), and JNK inhibitor SP600125 (10 uM, Selleck Chemicals, USA) (36) for 30 minutes before IL-15 (500pg/ml) treatment.

### Cell Counting Kit-8 Assay

Cell viability was determined by cell-counting kit-8 (CCK8) (Selleck, Shanghai, China). KGN cells and mouse primary GCs were seeded in 96-well plates at 1×103 cells/well in 100μl cell suspension. 10μl CCK-8 reagent was added to each well and then the cells were cultured for 1h at 37°C. Optical density was measured at 450 nm using a microplate reader (BioTeck, USA). Each experiment was carried out in triplicate at least.

### Apoptosis Assay

The apoptosis level of GCs cells was detected using Annexin V-FITC apoptosis kit (BD Pharmingen, San Diego, CA, USA) according to the manufacturer’s instructions. Experiments were performed by a CytoFLEX flow cytometer (Beckman coulter, Brea, CA, USA) and analyzed by FlowJo 10.0 software (Tree Star, Ashland, USA).

### RNA Isolation and Real-Time PCR (RT-PCR)

Total RNA was extracted from ovarian tissues and GCs using RNA-Trizol reagent (Invitrogen, Carlsbad, CA, USA). The RNA concentration of all samples was quantified by NanoDrop 1000 (Thermo Fisher Scientific, USA), and was reverse transcribed to cDNA by Reverse Transciption kit (Takara, Dalian, China). Quantitative reverse transcription PCR (RT-PCR) was performed in SYBR Green^®^ fast qPCR Mix (Takara, Dalian, China) on an ABI 7500 Real-Time PCR System (Applied Biosystems, Foster City, CA USA). Results were normalized using β-actin expression. Primers were designed using Primer-Blast tool by NCBI and present in **Supplementary Table 1**. Relative transcription levels were calculated using 2*^-ΔΔCT^* methods.

### Western Blot

Total protein was extracted using ice-cold radio-immunoprecipitation assay lysis buffer (Cwbio) containing a phosphatase inhibitor and a protease inhibitor cocktail (both from Roche). Protein from each sample was electro-phoresed in a 10% sodium dodecyl sulfate–polyacrylamide gel electrophoresis gel and then transferred onto a nitrocellulose blot. After 1 hour of blocking with 5% nonfat milk, the blot was incubated overnight at 4°C with antibodies against phospho-JNK (Thr183/Tyr185; 1:1000), and total JNK (1:1000), phospho-p38 MAPK (Thr180/Tyr182; 1:1000), total p38 MAPK (1:1000). On the second day, the blot was washed and then incubated with the respective secondary antibody conjugated to horseradish peroxidase (1:1500) for 1 hour. An enhanced chemiluminescent detection system (Millipore) was used to detect the bands with peroxidase activity. A G-Box iChemi Chemiluminescence image capture system (Syngene) was used to visualize the bands. The same blot was also probed with α-tubulin (1:1000) as internal controls. All antibodies were from Cell Signaling Technology (CST, Massachusetts, USA).

### GEO Data Analysis

Microarray datasets 155489 and GSE106724 were downloaded from Gene Expression Omnibus and collected using the following platforms: GPL20795 (HiSeq X Ten) and GPL21096 (Agilent-062918 Human lncRNA array V4.0).The raw data was converted to a recognizable format by GEO2R (https://www.ncbi.nlm.nih.gov/geo/geo2r/).

### Statistical Analyses

Statistical analysis was performed with GraphPad Prism version 8.0 software (GraphPad, San Diego, CA, USA), and data were presented as the mean± the standard error of the mean (SEM)or medians with interquartile ranges. Student’ t test of two independent samples was used for the data of normal distribution, and the Wilcoxon rank-sum test of two independent samples was used for the data of non-normal distribution. Pearson’s linear regression was used for correlation analysis to investigate the relationship among IL-15 and serum Testosterone concentration. Spearman’s or pearson’s correlation analysis were used to investigate the relationship among the relative expression of *IL-15* or *IL-2rg(IL-15r)*and *CYP17A1* in PCOS patients. The one-way ANOVA with Tukey’s multiple comparison *post-hoc* test was used for multiple groups. The *post hoc* statistical power value is expressed as “R”. A P<0.05 was considered statistically significant.

## Results

### Higher Level of IL-15 in the Follicular Fluid of Patients With PCOS

**Figure 1:**
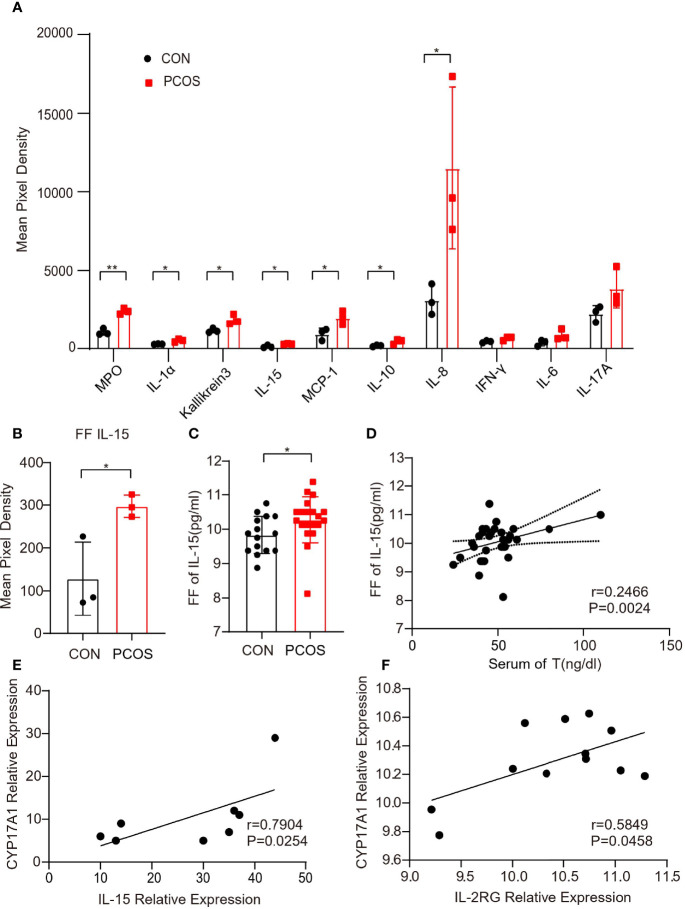
Up-regulation of IL-15 in the follicular fluid of PCOS patients. **(A)** Representative difference inflammation factors of follicular fluid factor chip between two groups of patients (n=3 per group, P=0.001, 0.017, 0.026, 0.031, 0.035, 0.040, 0.050; R=0.7924, 0.7924, 0.7476, 0.7280,0.7991, 0.6911, 0.6591 respectively); **(B)** Mean pixel density of IL-15 in follicular fluid of PCOS patients and non-PCOS controls (n=3 per group, P=0.045, R=0.523; **(C)** Follicular fluid of PCOS patients and controls ELISA level of IL-15 (PCOS n=20, CON n=15, P=0.0475, R=0.6018); **(D)** Correlation of IL-15 in follicular fluid levels with the serum levels of T as determined by Pearson’s rank test (P=0.0024, r=0.2466); **(E)** Correlation of relative expression of *IL-15* with *CYP17A1* in cumulus granulosa cells of PCOS patients and age-matched control (GSE155489) as determined by Spearman’s analysis (P=0.0254, r=0.7904); **(F)** Correlation of relative expression of *IL-2rg (IL-15r)* with *CYP17A1* in cumulus granulosa cells *o*f PCOS patients and non-PCOS women (GSE106724) as determined by Pearson’s analysis (P=0.0458, r=0.5849). *P < 0.05, **P < 0.01 versus the control.

In order to determine the alteration of cytokine and chemokine in the follicular fluid of PCOS women, we recruited 3 PCOS and 3 non-PCOS patients, whose clinical characteristics are illustrated in **Table 1**. In the PCOS patients, the BMI and serum LH, T and AMH level, and the ratio of LH/FSH were significantly higher, while the age, serum FSH and E2 levels were similar between the two groups. According to the findings of cytokine array, we found that chemokine MPO, IL-1α, Kallikrein3, IL-15, MCP-1, and IL-8 were significantly increased in PCOS group (**Figure 1A** and **Supplementary Figure 1A**). As previously reported, IL-15 is a regulator of obesity related pathological changes (37, 38), and can modulate insulin sensitivity (39). Whereas, obesity and insulin resistance (IR) are typical pathological changes in PCOS, we speculated that IL-15 plays an important role in PCOS. Then, the similar results were observed in FF collected from 20 PCOS and 15 non-PCOS patients (**Figure 1C**), and their clinical characteristics are illustrated in **Table 2**. Non-PCOS patients matched the BMI of PCOS patients, while the LH level of PCOS patients tends to be higher、the FSH level and E2 level tend to be lower and the T level is significantly higher compared with non-PCOS patients, which were also consistent with the clinical endocrine characteristics of PCOS. Furthermore, Pearson correlation analysis showed that the FF IL-15 concentration was positively correlated with serum T (r=0.2466, P = 0.0024, **Figure 1D**). And relative expression of *IL-15* was positively correlated with *CYP17A1* in cumulus granulosa cells of PCOS patients and age-matched control (r=0.7904, P=0.0254, **Figure 1E**) and relative expression of *IL-2rg (IL-15r)* was positively correlated with *CYP17A1 in* cumulus granulosa cells of PCOS patients and non-PCOS women (r=0.5849, P=0.0458, **Figure 1F**) by using bioinformatics analysis.

### Higher Level of IL-15 in PCOS Model Mice

**Figure 2:**
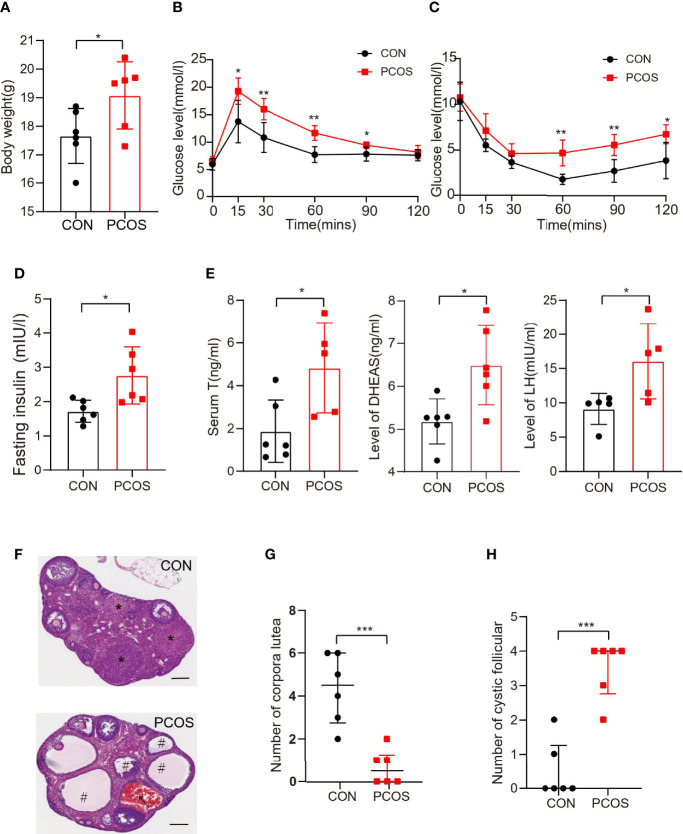
The phenotypes of the PCOS model mice are similar to those of the PCOS patients. The mice that implanted with letrozole sustained-release tablets and control mice were defined as CON and PCOS, respectively. **(A)** The weight of the two groups after three weeks (n=6 per group, P=0.0455, R=0.3428); **(B)** GTT (n = 6 mice per group, for 15 mins, P=0.0142, R=0.3428; for 30 mins, P=0.0042, R=0.7891; for 60 mins, P=0.0013, R=0.8018; for 90 mins, P=0.0200, R=0.4914). **(C)** ITT (n = 6 mice per group, for 60 mins, P=0.0013, R=0.8924; for 90 mins, P=0.0028, R=0.7614; for 120 mins, P=0.0113, R**=**0.5518); **(D)** The level of fasting insulin in two groups (n=6 mice per group, P=0.014,R=0.4517); **(E)** The level of T、DHEAS and LH in two groups(for the level of T, CON=6, PCOS=5 , P=0.0223, R=0.4577; for the level of DEHAS, n=6 per group, P=0.0129, R=0.4771; for the level of LH, n=5 per group, P=0.0308, R=0.4611); **(F)** Hematoxylin and eosin staining of representative ovaries. The cystic follicle is indicated by a hashtag, while the corpora lutea are indicated by asterisks. Arrow indicates serum cyst. Scale bar: 200 μm. **(G, H)** The number of corpora lutea and cystic follicle in two groups (n = 6  per group; P=0.0006, 0.0001; R=0.7076, 0.7941 respectively). *P < 0.05, **P < 0.01, ***P < 0.001 versus the control.

In order to verify the role of IL-15 in the pathogenesis of PCOS, the well-established LTZ-induced PCOS mouse model was confirmed. The mice in the PCOS group were significantly heavier compared with age-matched control mice that had received placebo pellets (**Figure 2A**). Similar results were observed in the weight of ovary and fat pads in PCOS group (**Supplementary Figure 1C, D**). PCOS mice had significantly reduced glucose tolerance and insulin sensitivity (**Figures 2B, C**). And we tested the fasting blood glucose of the mice and calculated HOMA-IR. The results showed that the PCOS mice had obvious insulin resistance compared with the control group (**Figure 2D** and **Supplementary Figure 1E**). PCOS mice also displayed an irregular estrous cycle (**Supplementary Figure 1F**). The levels of serum T, DHEAS and LH were significantly higher in PCOS mice (P<0.05) compared with the control, but there is no significant difference in serum FSH levels (**Figure 2E** and **Supplementary Figure 1G**). Atresia and cystic dilated follicles were significantly increased and the number of corpora lutea was decreased in the PCOS mice compared with the control mice (**Figures 2F–H**), indicating similar ovarian dysfunction to the clinical presentation of PCOS.

**Figure 3:**
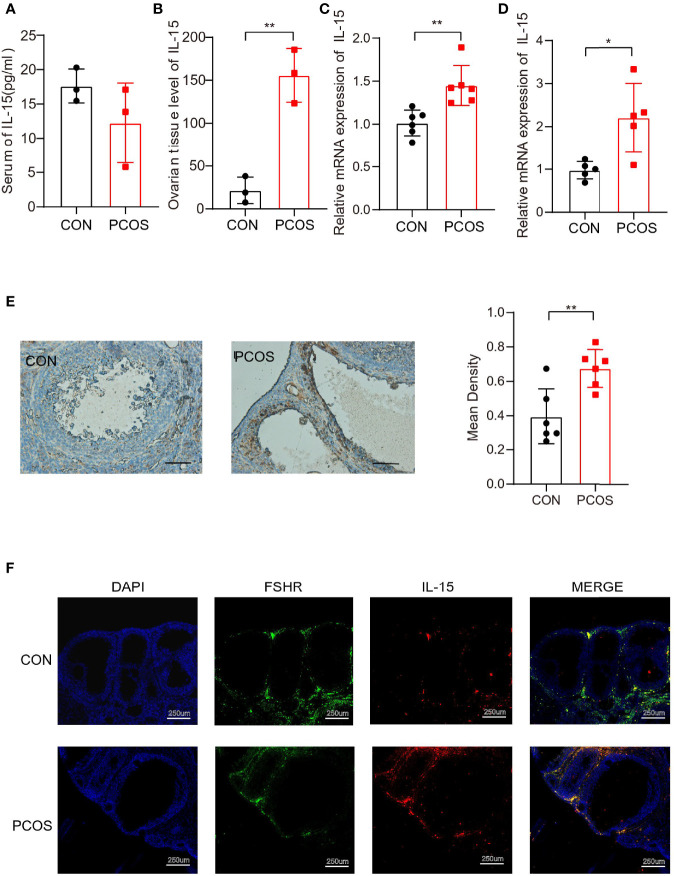
Higher level of IL-15 in PCOS model mice. **(A, B)**. ELISA detection of IL-15 in the serum and ovarian tissue homogenate of two groups of mice (n=3 per group, P=0.2146, 0.0026; R=0.3044, 0.9179 respectively); **(C)** PCR detection of IL-15 in ovarian tissues in the two groups (CON n=5, PCOS n=6, P=0.0032, R=0.5984); **(D)** PCR detection of IL-15 in adipose tissue in the two groups (n=6 per group, P=0.0103, R=0.5819); **(E)** IHC analysis of IL-15 in the ovaries of two groups of mice. The brown part represents the part of IL-15 expression. Bar=50um, 40X. Images are representative of three independent experiments with similar results (P=0.0055, R=0.5541); **(F)**, Blue shows the nucleus (DAPI), green fluorescence shows ovarian granulosa cells marker FSHR (TSA 520, excitation and emission wavelengths similar to FITC), red shows IL-15(TSA 650, excitation and emission wavelengths similar to AF610). Bar=250um. *P < 0.05, **P < 0.01 versus the control.

Consistently, we found that IL-15 mRNA and protein expression were elevated significantly in ovarian of PCOS mice compared with control, while the serum IL-15 level was similar between two groups (**Figures 3A–C**). Moreover, the IL-15 mRNA expression was elevated significantly in adipose tissues of PCOS mice compared with control, suggesting that IL-15 may play a role in chronic inflammation of adipose (**Figure 3D**). IHC results confirmed that IL-15 was overexpressed in PCOS mice compared with control (**Figure 3E**), and colocalized with FSHR, a GCs marker (**Figure 3F**). This data suggested that IL-15 may be involved in the pathogenic role of GCs in PCOS.

### Effects of IL-15 on the Proliferation and Apoptosis of KGN Cells

**Figure 4:**
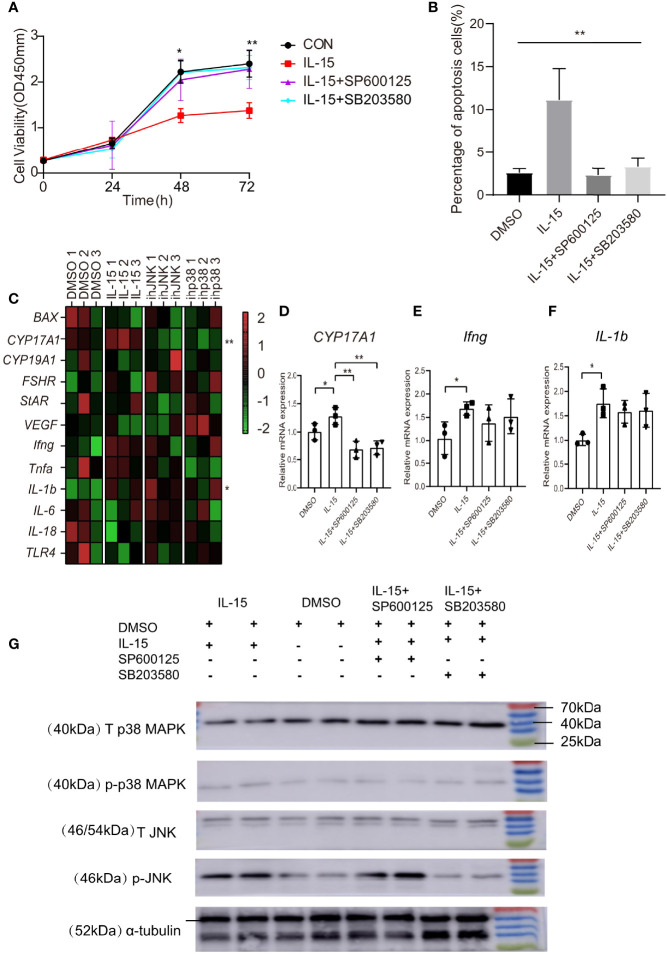
IL-15 inhibits the proliferation of KGN, promotes its apoptosis and dysfunction. **(A)** Effects of IL-15 and inhibitors on the cell viability was assessed by CCK-8 assay(for 48h, P=0.0124, R=7252; for 72h, P=0.0097, R=0.7423; **(B)** Flow cytometric analysis of apoptosis cells (Annexin V+ cells). Percentage of Annexin V+ cells in mutiple groups under different conditions (n = 3 per group, P=0.0015, R=0.8399); *P < 0.05, **P < 0.01 in multiple groups by one-way ANOVA; **(C)** Heat map of inflammatory factor gene and functional gene expression in KGN cell line after treatment. Relative expression of genes were transformed into Z-score maps based on mean and SD values(for *CYP17A1*, P=0.0031, R=0.8079; for *IL-1b*, P=0.0356,R=0.6379); **(D–F)** relative expression of *CYP17A1、Ifng* and *IL-1b* in different conditions(n=3 per group, for *CYP17A1: *P=0.0472, 0.0026, 0.0035 respectively compared with IL-15; for *Ifng*, P=0.0442; for *IL-1b*, P=0.0281), *P < 0.05, **P < 0.01 versus IL-15.; **(G)** Representative western blot analysis and densitometric analysis of total p38 MAPK (T p38 MAPK)、 total JNK (T JNK)、phospho-p38 MAPK (p-p38 MAPK)、phospho-JNK (p-JNK) and α-tubulin in KGN cells treated with different conditions.

Due to the significant association of IL-15 with the clinic pathological characteristics in PCOS women and the colocalization between IL-15 and GCs in PCOS mice, we examine the effects of IL-15 on GCs growth and apoptosis. Our data showed that IL-15 suppressed proliferation of KGN cells and the intervention of JNK inhibitor SP600125 and p38 MAPK inhibitor SB203580 alleviated this effect of IL-15 (**Figure 4A**). The results obtained by flow cytometry analysis are also consistent with CCK-8 assay (**Figure 4B**). These results suggested that IL-15 decreased proliferation, whereas increased apoptosis in GCs.

### Effects of IL-15 on Steroidogenesis and the State of Inflammation in KGN Cells

To delineate the role of IL-15 in GCs steroidogenesis and the state of inflammation, we cultured the KGN cells in the presence or absence of IL-15 with or without inhibitors. Treatment of KGN cells with IL-15 for 4 h resulted in significantly increases the mRNA levels of cytochrome P450, family 17, subfamily A, polypeptide 1 (*CYP17A1*), which converts progesterone to 17α-hydroxyprogesterone and androstenedione (**Figures 4C, D**). Interestingly, we found that these two inhibitors have inhibitory effects on the increase in *CYP17A1* expression induced by IL-15. Consistent to previous studies that IL-15 function as a proinflammatory cytokine (40), IL-15 significantly increased mRNA level of *IL-1b* and *Ifng* and the above two inhibitors have a tendency to reverse this effect (**Figured 4E, F**). These data suggested that IL-15 might play a role in the pathogenesis of PCOS by increasing production of androgen hormones and sustaining inflammation state in GCs, and these effects may be related to the activation of the signaling pathways p38 MAPK and JNK. Therefore, we carried out the detection of these two signaling pathway molecules and their phosphorylation levels on different treated KGN cells by Western Blot. The results of WB showed that IL-15 treatment increased the phosphorylation level of JNK and P38 MAPK in the KGN cell line compared with DMSO alone (**Figure 4G**).

### Effects of IL-15 on the Proliferation and Apoptosis of Mouse Primary Granulosa Cells

**Figure 5:**
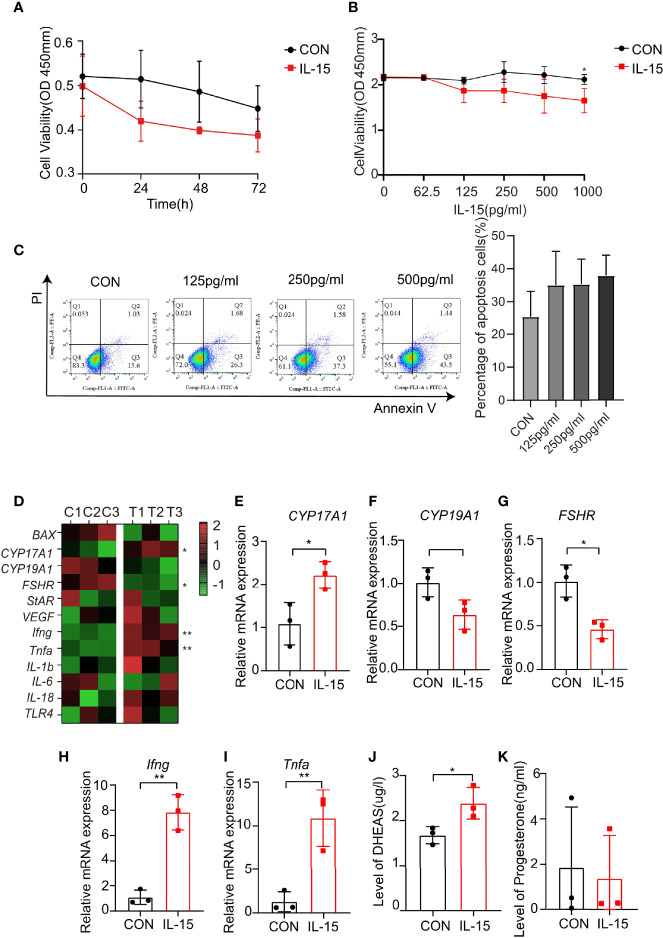
IL-15 inhibits the proliferation of mGCs and promotes its apoptosis and dysfunction. **(A, B)** Time-and dose-dependent effects of IL-15 on the mGCs was viability assessed by CCK-8 assay (n=3, *P=0.0356, R=0.6627); **(C)** Flow cytometric analysis of apoptosis cells (Annexin V+ cells), three independent experiments were performed with similar results. Percentage of Annexin V+ cells in two groups under different conditions (n = 3 per group); **(D)** Heat map of inflammatory factor genes and functional gene expression in primary cell lines after treatment. Control group: C1, C2, C3; IL-15 treatment group: T1, T2, T3. Relative expressions of genes were transformed into Z-score maps based on mean and SD values; **(E–I)** relative expression of *CYP17A1, FSHR, Ifng* and *Tnfa* in two groups(P=0.0278, 0.0114, 0.0015, 0.0083;R=0.7405, 0.8306, 0.9381, 0.8551 respectively); **(J, K)** DHEAS and P levels of the culture supernatant (n=3 per group, *P=0.0457, R=0.4527). *P < 0.05, **P < 0.01 versus the control.

IL-15 suppressed proliferation of primary GCs cells at dose dependent manner (**Figures 5A, B**). In addition, IL-15 promoted apoptosis of primary mGCs at dose dependent manner (**Figure 5C**).

### Effects of IL-15 on Steroidogenesis and the State of Inflammatory in Mouse Primary Granulosa Cells

Consistent with previous study, IL-15 significantly increased mRNA levels of *CYP17A1*, while decreased mRNA levels of *CYP19A1* and *FSHR* (**Figures 5D–G**). IL-15 also increased proinflammatory cytokine mRNA levels of *Ifng* and *Tnfa* (**Figures 5H, I**). The effects of IL-15 on the steroidogenic activity of primary GCs were evaluated. The level of DHEAS and progesterone in the medium secreted from primary GCs for 24 h in the presence of IL-15 were measured. The concentration of DHEAS were significantly increased after incubation of the cells with IL-15 (**Figure 5J**), while the concentration of progesterone remained unaltered (**Figure 5K**). These data confirmed the results obtained from the human GCs cell line KGN.

## Discussion

Ovarian granulosa cells (GC) are important somatic cells in ovarian tissue which are stimulated by FSH. They secrete insulin-like growth factors and promote the development of follicles. In addition, various aromatase families can promote cholesterol metabolism and secrete synthetic sex hormones, which play an important role in female reproductive health (41). In this study, we evaluated the effects of IL-15 on GC functions including GC proliferation, expression of inflammatory factors and steroidogenesis, aiming to examine the potential involvement of IL-15 in regulating ovarian follicle function by utilizing the KGN cells and a primary mouse granulosa cells culture model. And we found that IL-15 affects the proliferation and function of GC through p38 MAPK and JNK phosphorylation.

Chronic inflammation is an important pathogenic factor of PCOS. In the analysis of the cytokine and chemokine array of the patient’s follicular fluid, we found that IL-15 significantly elevated in PCOS group. And except it, we found that MPO, IL-1α, Kallikrein3, MCP-1, and IL-8 significantly elevated in PCOS group, proving that the ovarian of PCOS patients does have a chronic inflammation state. Previous studies reported that these inflammatory factors are associated with insulin resistance and promoting androgen release (42–46). However, these inflammatory factors except IL-8 have been reported to be elevated only in the serum of PCOS patients before, the results of our follicular fluid provide ideas for studying the relationship between circulatory inflammation and ovarian inflammation in patients with PCOS.

We found that the level of follicular fluid (FF) IL-15 was higher than that in the control mice group, which was consistent with the results of PCOS patients. But the level of IL-15 in serum didn’t significant increase. The results seem to indicate that IL-15 is produced locally in the ovarian tissue, or certain factors may promote the accumulation of IL-15 in the ovarian tissue. By co-localizing mouse granulosa cells and IL-15 immunofluorescence, we found that the position of IL-15 and granulosa cells are highly co-localized, indicating that granulosa cells are the main somatic cells that produce IL-15 in ovarian tissue, but which factors lead to increased IL-15 secretion by ovarian granulosa cells need further research. In addition, we also found that the expression level of IL-15 in adipose tissue was significantly up-regulated in the PCOS model mouse group. Studies have shown that the adipose tissue of patients with metabolic abnormalities such as obesity is in a state of chronic inflammation (47). The results suggested that the inflammatory reaction occurred not only in the FF but also in the fat tissue of the PCOS.

IL-15 is generally regarded as a T cell growth factor and is belongs to the cytokine receptor γ chain (γc) family. It shows a wide range of pleiotropic effects regulating the innate and adaptive immune system, regulates cell differentiation and promoting survival or inducing apoptosis according to the cell environment (48). But there is no research showing that IL-15 has a direct effect on the apoptosis of somatic cells. The present studies provide new insights into the intracellular signaling cascade by which IL-15 induces GCs apoptosis. Our research showed that IL-15 not only induced *Ifng* but also *Tnfa* expression. Both IFN-γ and TNF-α promote cell apoptosis. IFN-gamma(IFN-γ) modulates the apoptotic pathway by upregulating apoptosis-related genes and overproduction of pro-inflammatory cytokine IFN-γ induces the excessive apoptosis of IECs and is involved in Crohn’s disease development (49, 50). And studies have shown that IFN-γ can promote the apoptosis of ovarian granulosa cells (51). TNF-α can induce hepatocyte apoptosis and liver damage (52). And our study found that IL-15 promoted the increase of mGCs synthesis DHEAS. Study have shown that the increase of androgens inhibits the proliferation of granulosa cells (53), so the pro-apoptotic effect of IL-15 on ovarian granulosa cells may be indirectly achieved by promoting the androgen and inflammation state of follicular fluid microenvironment. Follicular atresia in PCOS patients is associated with increased granulosa cell apoptosis (54). Our study revealed that high doses of IL-15 inhibited the proliferation of GCs, indicating that the increase in granuloma cells apoptosis observed under pathological conditions (such as PCOS) may be caused by IL-15 or the immune microenvironmental changes caused by IL-15 stimulation. Therefore, based on these findings, we believe that IL-15 participates in the follicular atresia of PCOS by promoting the apoptosis of granulosa cells.

Elevated androgen is an important pathological manifestation of PCOS. We found that treatment of IL-15 increases the expression of *CYP17A1* in granulosa cells. CYP17A1 is a critically important enzyme in humans that catalyzes the formation of androgens. It catalyzes the 17α-hydroxylation of pregnenolone to 17α-OH pregnenolone. Subsequently, through its C17,20 lyase activity, it can further convert 17α-OH pregnenolone to the androgen dehydroepiandrosterone, which is the precursor of androstenedione, testosterone and dihydrotestosterone (55). The increase in DHEAS of the culture supernatant of mGCs treated with IL-15 also indicates that IL-15 promotes the secretion of androgens from granulosa cells. IL-15 forms a complex with receptors IL-2Rβ and γ chain (IL-2Rγ) through IL-15Rα, and completes the process of signal transduction to play immune and other functions (56). Our analysis of the database showed that the expression of *IL-15, IL-2rg* and *CYP17A1 in granulosa cells of PCOS patients* were positively correlated, indicating that IL-15 was involved in androgen synthesis. And there is a randomized controlled trial using BNZ-1, a selective and simultaneous inhibitor of cytokines IL-2, IL-9, and IL-15, for the treatment of hyperandrogen-induced hair loss (57).It shows that factors that inhibit IL-15 such as BNZ-1 have a role in the treatment of PCOS hyperandrogenism.

CYP19A1 is considered to be an important marker in the etiology of polycystic ovary syndrome. The study reported that compared with healthy controls, the aromatase gene expression in PCOS follicles before ovulation and the subsequent estradiol production decreased (58). And it can convert androgen into estrogen (59). FSHR plays an important role in synthesizing estrogen and stimulating follicular development (60, 61). IL-15 treatment reduced the expression of *CYP19A1*, and *FSHR* both in mGCs. Those all indicated that IL-15 can affect follicle development by influence hormone synthesis related enzymes and participate in the follicular atresia of PCOS.

We found that IL-15 promoted the phosphorylation of p38 MAPK and JNK in KGN cells, and the addition of corresponding inhibitors would reverse the effects of IL-15 on the proliferation inhibition and functional genes and expression of pro-inflammatory factors in KGN cell lines. It reminds us that IL-15 may play a role through these two signaling pathways. This needs to be further verified in mGCs.

For the first time, we reported that IL-15 decreased KGN cells and mGCs proliferation as well as key genes for follicular development such as *CYP19A1* and *FSHR*. IL-15 also up-regulated the expression of *CYP17A1, the* key steroidogenic-related genes in androstenedione secretion. In addition, IL-15 can increase the expression of pro-inflammatory factor *IL-6*, *Tnfa* and *Ifng* of granulosa cells, and these pro-inflammatory factors have been reported to be related to the metabolism of PCOS and the abnormal reproductive phenotype, and promote the chronicity of the microenvironment around the follicle. And this effect is through p38 MAPK and JNK phosphorylation. The present study provides new evidence for IL-15-dependent regulation of proliferation and steroidogenesis in GCs that may influence follicle development. It is now convincingly suggested that IL-15 may serve as a critical regulator of cell proliferation, differentiation and steroidogenesis in the KGN and mGCs of ovarian preantral follicle. But additional research is needed to understand the mechanism of action of IL-15 in granulosa cells, as well as its exact role in follicle development. The current clinical treatment and efficacy evaluation of PCOS, due to inconsistent diagnostic criteria, patients often have repeated symptoms. IL-15, as a pro-inflammatory factor, participates in the pathogenic process of PCOS, showing the potential in evaluation of the diagnosis and treatment effect of PCOS. However, this study only analyzed the signal pathways of the human KGN cell line, did not study mGCs and did not conduct IL-15 intervention experiments and rescue experiments *in vivo*. The sample size of patients used to detect IL-15 is relatively small. We will continue to improve in the future.

Further efforts are needed to demonstrate a connection between inflammatory factors and PCOS pathogenesis. Research on which signaling pathway is used by IL-15 to promote the secretion of androgens by granulosa cells needs to be further explored. By verifying the involvement of inflammatory factors in the pathogenesis of PCOS, new therapy may be developed for PCOS treatment, such as blocking therapeutic targets including inflammatory factors and signaling pathways.

## Data Availability Statement

The raw data supporting the conclusions of this article will be made available by the authors, without undue reservation.

## Ethics Statement

The studies involving human participants were reviewed and approved by the Shanghai Jiai Genetics and Infertility Diagnosis and Treatment Center Assisted Reproductive Ethics Committee. The patients/participants provided their written informed consent to participate in this study. The animal study was reviewed and approved by the ethics committee of Fudan University.

## Author Contributions

YL designed the experiments, acquired, analyzed, and interpreted the data, and drafted the manuscript. ZL contributed to the experiment. HL, CX, and FZ provided substantial contributions to the conception of the study, experimental design, and data interpretation. All authors revised and approved the final version of the manuscript.

## Funding

This work was supported by the Shanghai Municipal Commission of Health and Family Planning (201640362) to FZ; the Shanghai Municipal Commission of Health and Family Planning (2017ZZ01016) to CX.

## Conflict of Interest

The authors declare that the research was conducted in the absence of any commercial or financial relationships that could be construed as a potential conflict of interest.

## Publisher’s Note

All claims expressed in this article are solely those of the authors and do not necessarily represent those of their affiliated organizations, or those of the publisher, the editors and the reviewers. Any product that may be evaluated in this article, or claim that may be made by its manufacturer, is not guaranteed or endorsed by the publisher.

## Supplementary Material

Supplementary Figure 1
**(A)** The alignment of 102 cytokines on the Human XL Cytokine Array Kit, R&D Systems. RS, reference spots; Acrp30, adiponectin; ApoA1, apolipoprotein A-1; Ang-1, angiopoietin-1; Ang-2, angiopoietin-2; BLyS, blymphocytestimulator; BDNF, brain-derived neurotrophic factor; CHI3L1, Chitinase 3-like 1; CFD, complement factor D; CRP, C-reactive protein; Cripto-1, teratocarcinoma-derived growth factor; Dkk-1, dickkopf-1; DPPIV, ipeptidyl-peptidase IV; EGF, epidermal growth factor; FGF-2, fibroblast growth factor 2; FGF-7, fibroblast growth factor 7; FGF-19, fibroblast growth factor 19; FLT3LG, Fms-related tyrosine kinase 3 ligands; G-CSF, granulocyte colony-stimu1ating factor; GDF-15, growth/differentiation factor 15; GM-CSF, granulocyte-macrophage colony stimulating factor; GH, growth hormone; SF, scatter factor; IGFBP-2, insulin-like growth factor binding protein 2; IGFBP-3, insulin-like growth factor binding protein 3; LIF, leukemia inhibitory factor; M-CSF, macrophage colony stimulating factor; MIF, macrophage migration inhibiting factor; MMP-9, matrix metalloprotein 9; MPO, myeloperoxidase; OPN, osteopontin; PDGF-AA, platelet-derived growth factor AA; PDGF-AB/BB, platelet-derived growth factor AB/BB; PTX3, pentraxin 3; RBP-4; retinol binding protein 4; SHBG, sex hormone-binding globulin; TFF-3, trefoil factor 3; THBS1, thrombospondin-1;uPAR, urokinase-type plasminogen activator receptor; VEGF, vascular endothelial growth factor; VDBP, vitamin D BP; TIM-3, T cell immunoglobulin domain and mucin domain-3; VCAM-1, vascular cell adhesion protein 1; NC, negative controls. **(B)** Representative images of cytokine array blots probed with the follicular fluid samples. Each blot represents immunoreactive staining against respective antibodies. Note the absence of staining at the negative control and blank slots. The relative expression levels of each cytokine were determined by comparing the pixel intensity of the respective blots to that of the positive control on the same array. The blots marked with red box are the cytokines that were significantly regulated in PCOS group compared to the non-PCOS group; **(C)**, The ovarian weight of the two groups of mice (n=6 per group); **(D)**, The fat pad weight of the two groups of mice (n=6 per group, P=0.0012, R=0.6681); **(E)**, HOME-IR in two groups (n=6 per group, P=0.0500, R=0.3317), **(F)**, Representative estrous cycles. Y-axis: P, proestrus; E, estrus; M, metestrus; D, diestrus; **(G)**, Serum FSH levels in mice.*P < 0.05 versus the control.

Supplementary Table 1 Primer sequence list.
