# Supplementary figures and images for: IL-15 Participates in the Pathogenesis of Polycystic Ovary Syndrome by Affecting the Activity of Granulosa Cells
Source: Front Endocrinol (Lausanne). 2022 Feb 18;13:787876. doi: 10.3389/fendo.2022.787876 (PMC8894602; doi:10.3389/fendo.2022.787876)

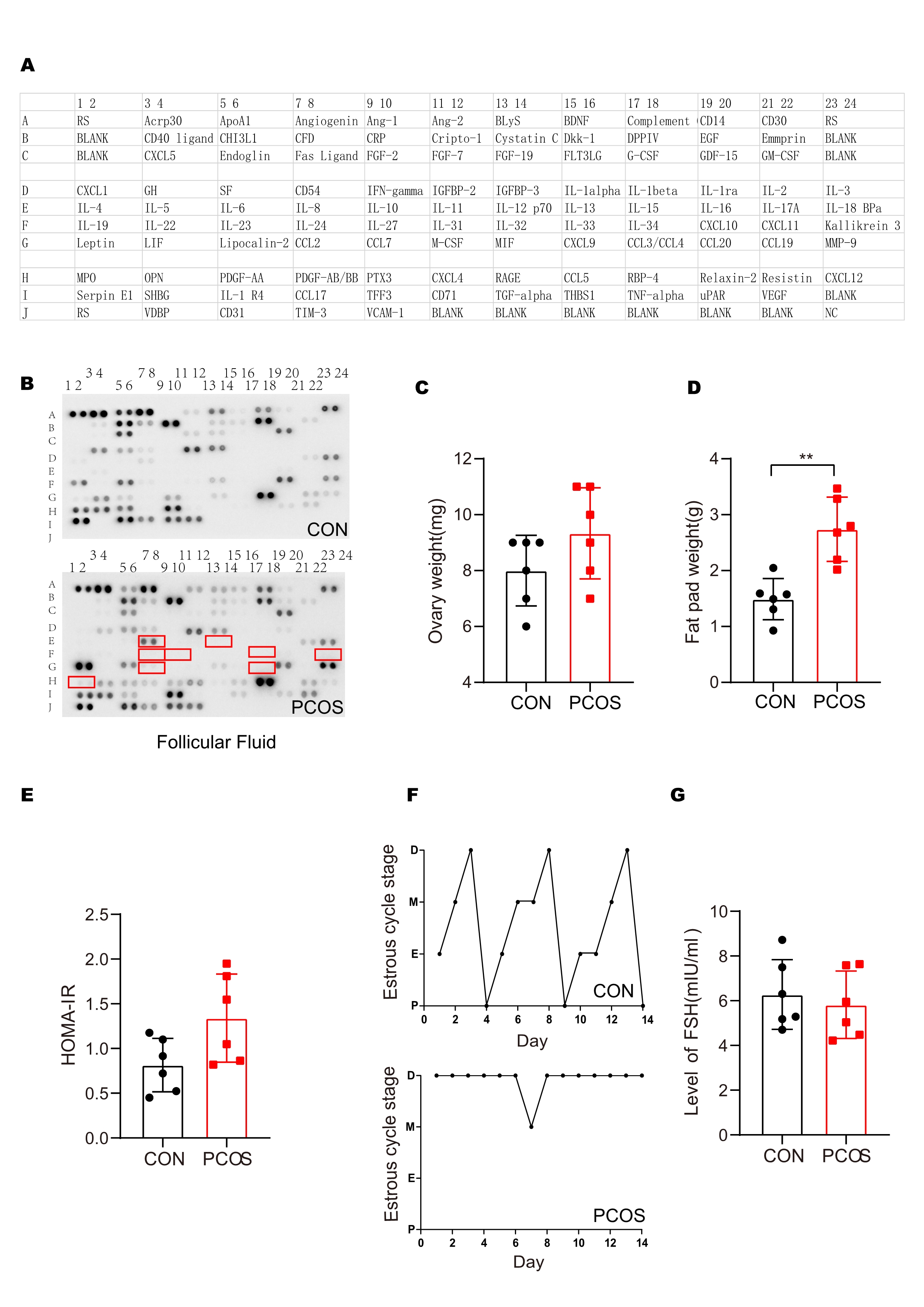

Supplement: Supplementary Figure 1 — (A) The alignment of 102 cytokines on the Human XL Cytokine Array Kit, R&D Systems. RS, reference spots; Acrp30, adiponectin; ApoA1, apolipoprotein A-1; Ang-1, angiopoietin-1; Ang-2, angiopoietin-2; BLyS, blymphocytestimulator; BDNF, brain-derived neurotrophic factor; CHI3L1, Chitinase 3-like 1; CFD, complement factor D; CRP, C-reactive protein; Cripto-1, teratocarcinoma-derived growth factor; Dkk-1, dickkopf-1; DPPIV, ipeptidyl-peptidase IV; EGF, epidermal growth factor; FGF-2, fibroblast growth factor 2; FGF-7, fibroblast growth factor 7; FGF-19, fibroblast growth factor 19; FLT3LG, Fms-related tyrosine kinase 3 ligands; G-CSF, granulocyte colony-stimu1ating factor; GDF-15, growth/differentiation factor 15; GM-CSF, granulocyte-macrophage colony stimulating factor; GH, growth hormone; SF, scatter factor; IGFBP-2, insulin-like growth factor binding protein 2; IGFBP-3, insulin-like growth factor binding protein 3; LIF, leukemia inhibitory factor; M-CSF, macrophage colony stimulating factor; MIF, macrophage migration inhibiting factor; MMP-9, matrix metalloprotein 9; MPO, myeloperoxidase; OPN, osteopontin; PDGF-AA, platelet-derived growth factor AA; PDGF-AB/BB, platelet-derived growth factor AB/BB; PTX3, pentraxin 3; RBP-4; retinol binding protein 4; SHBG, sex hormone-binding globulin; TFF-3, trefoil factor 3; THBS1, thrombospondin-1;uPAR, urokinase-type plasminogen activator receptor; VEGF, vascular endothelial growth factor; VDBP, vitamin D BP; TIM-3, T cell immunoglobulin domain and mucin domain-3; VCAM-1, vascular cell adhesion protein 1; NC, negative controls. (B) Representative images of cytokine array blots probed with the follicular fluid samples. Each blot represents immunoreactive staining against respective antibodies. Note the absence of staining at the negative control and blank slots. The relative expression levels of each cytokine were determined by comparing the pixel intensity of the respective blots to that of the positive control on the same array. [file Image_1.jpeg]

CON


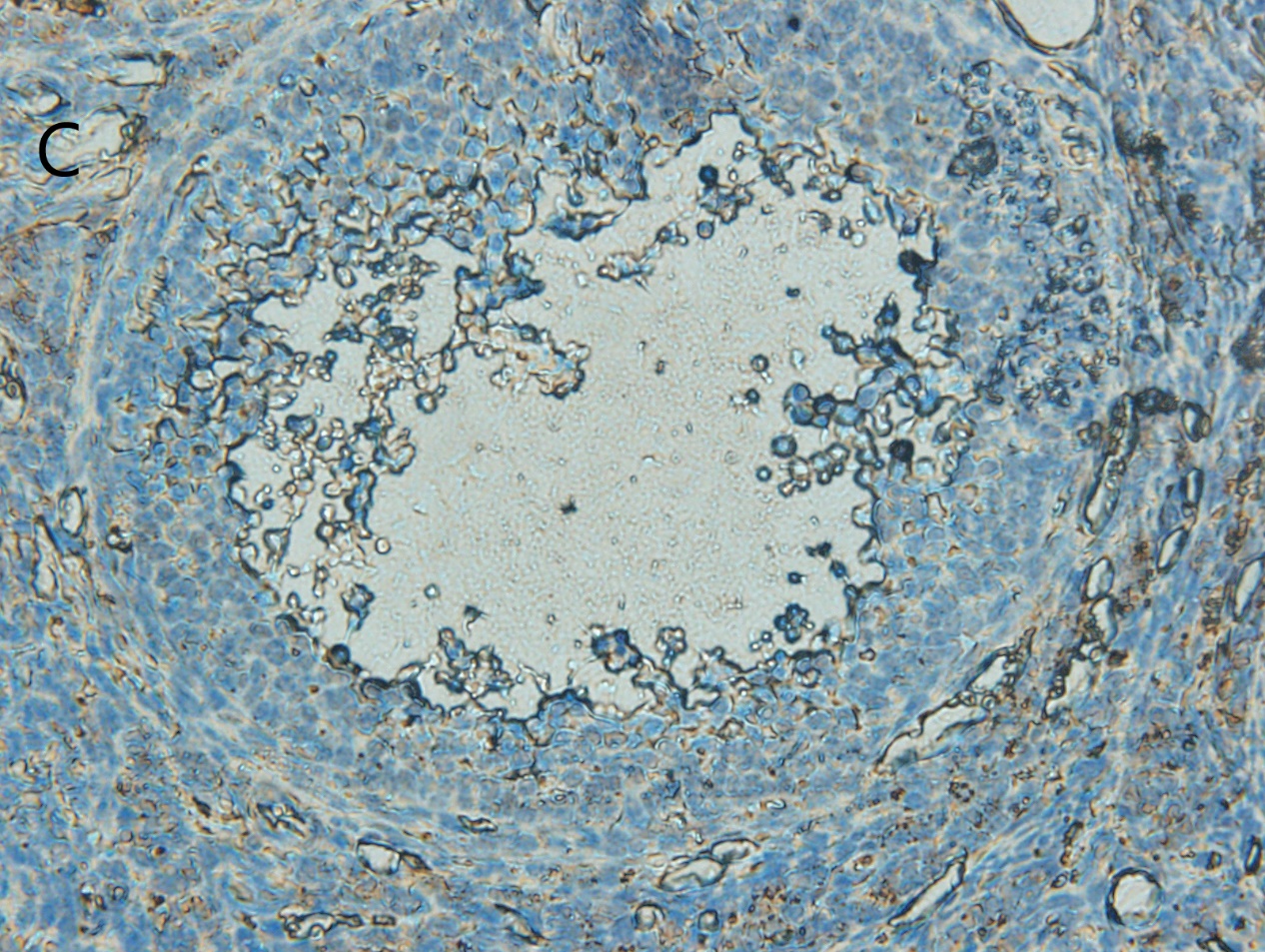


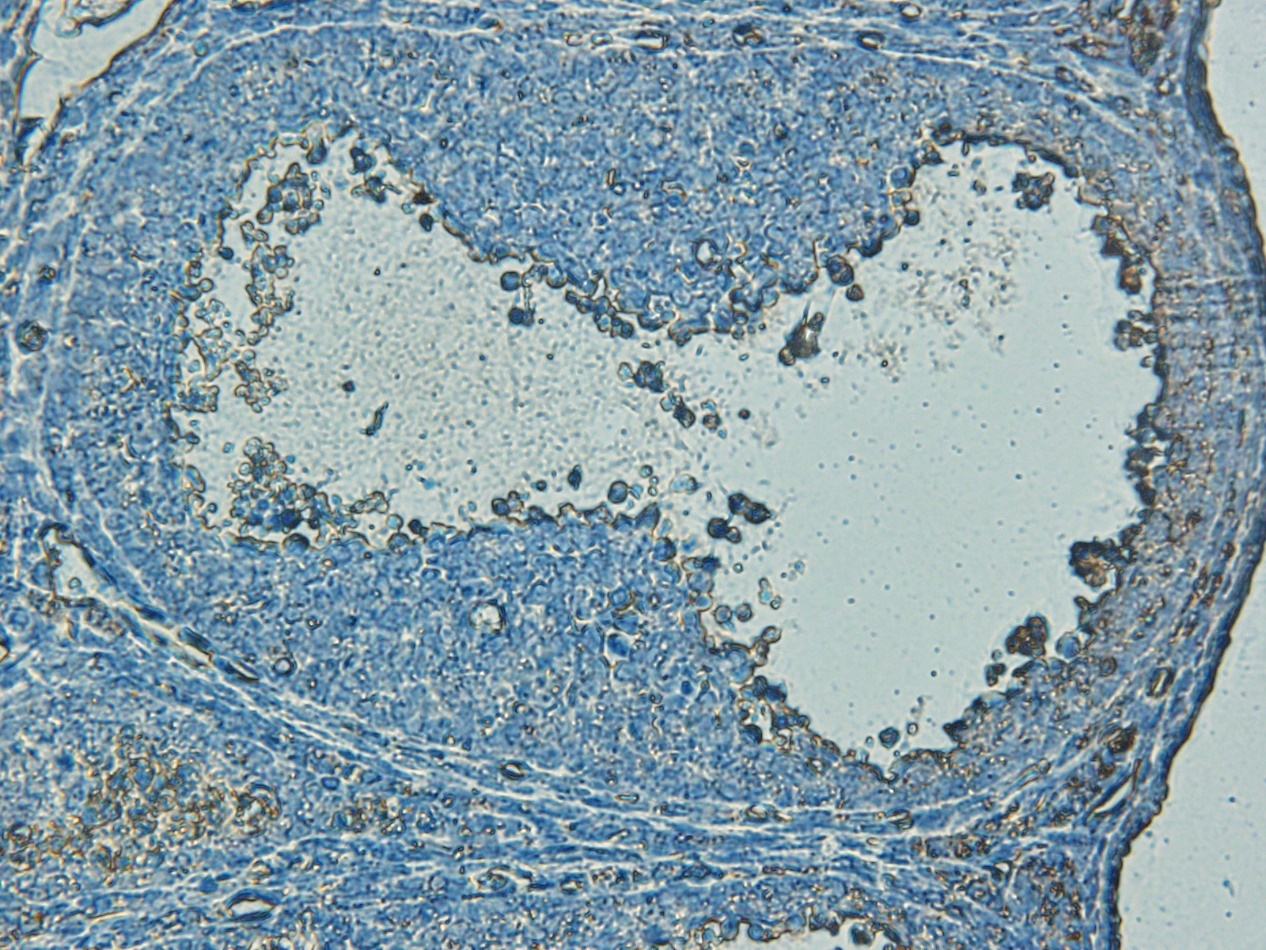


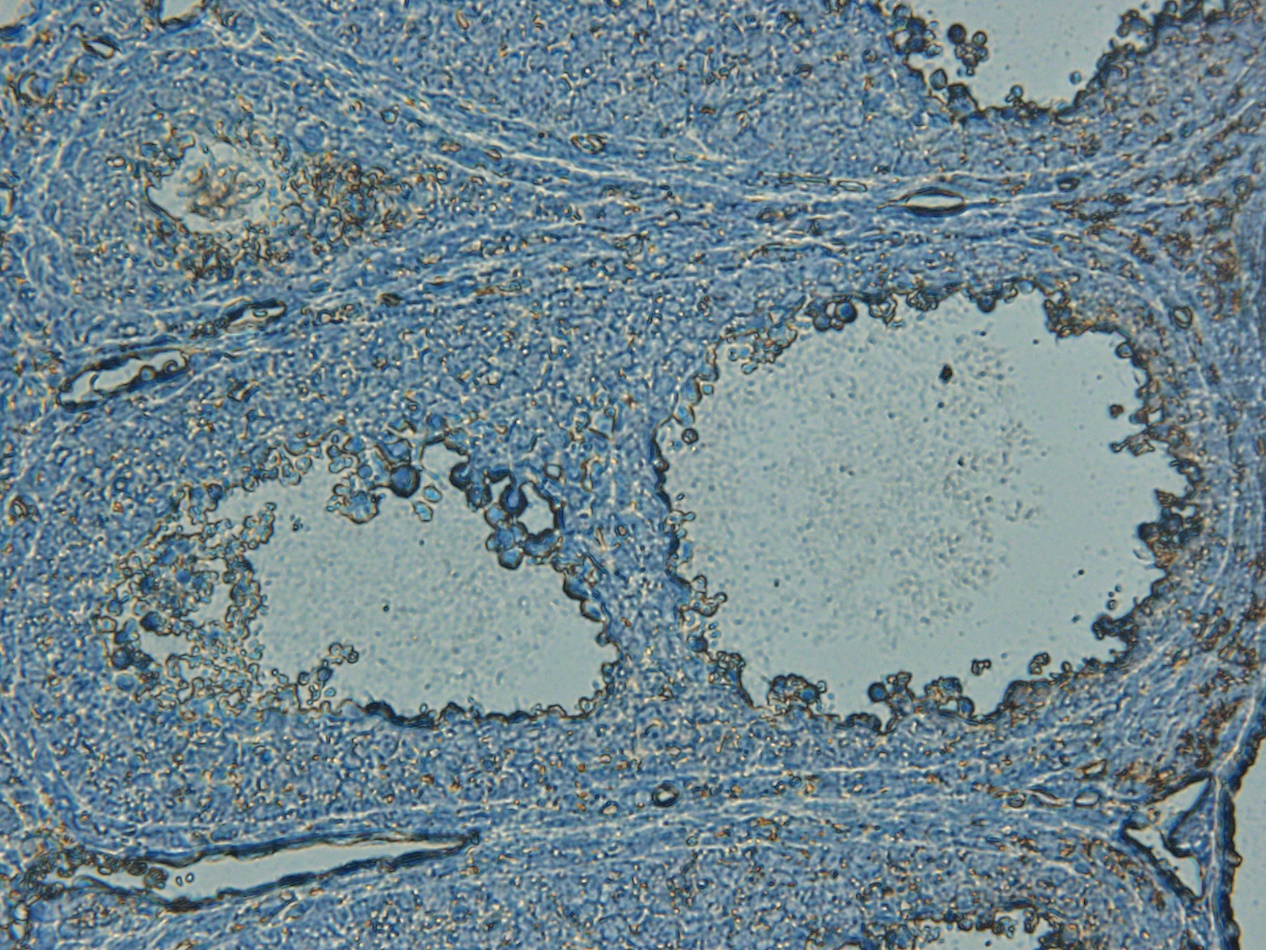


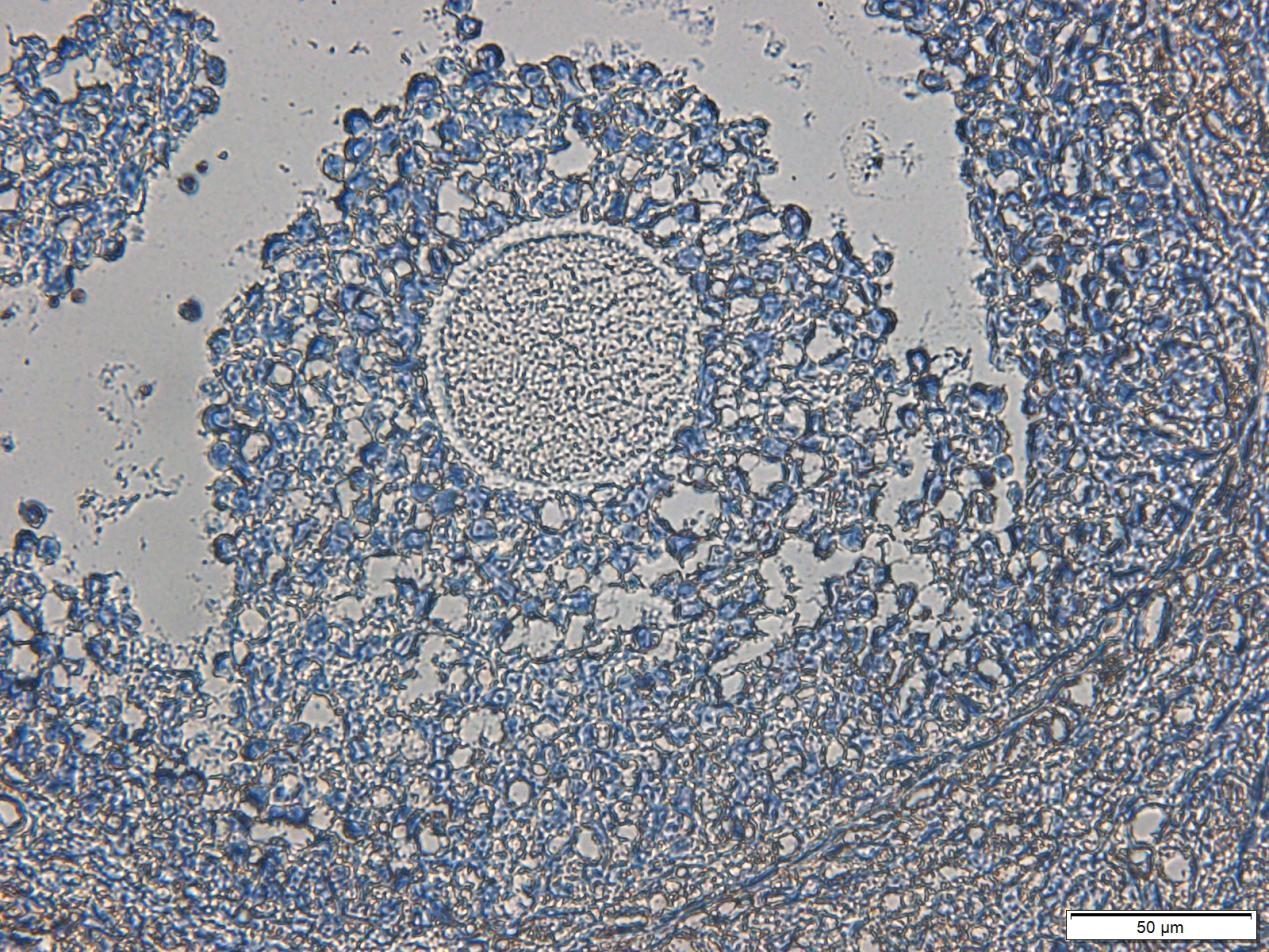


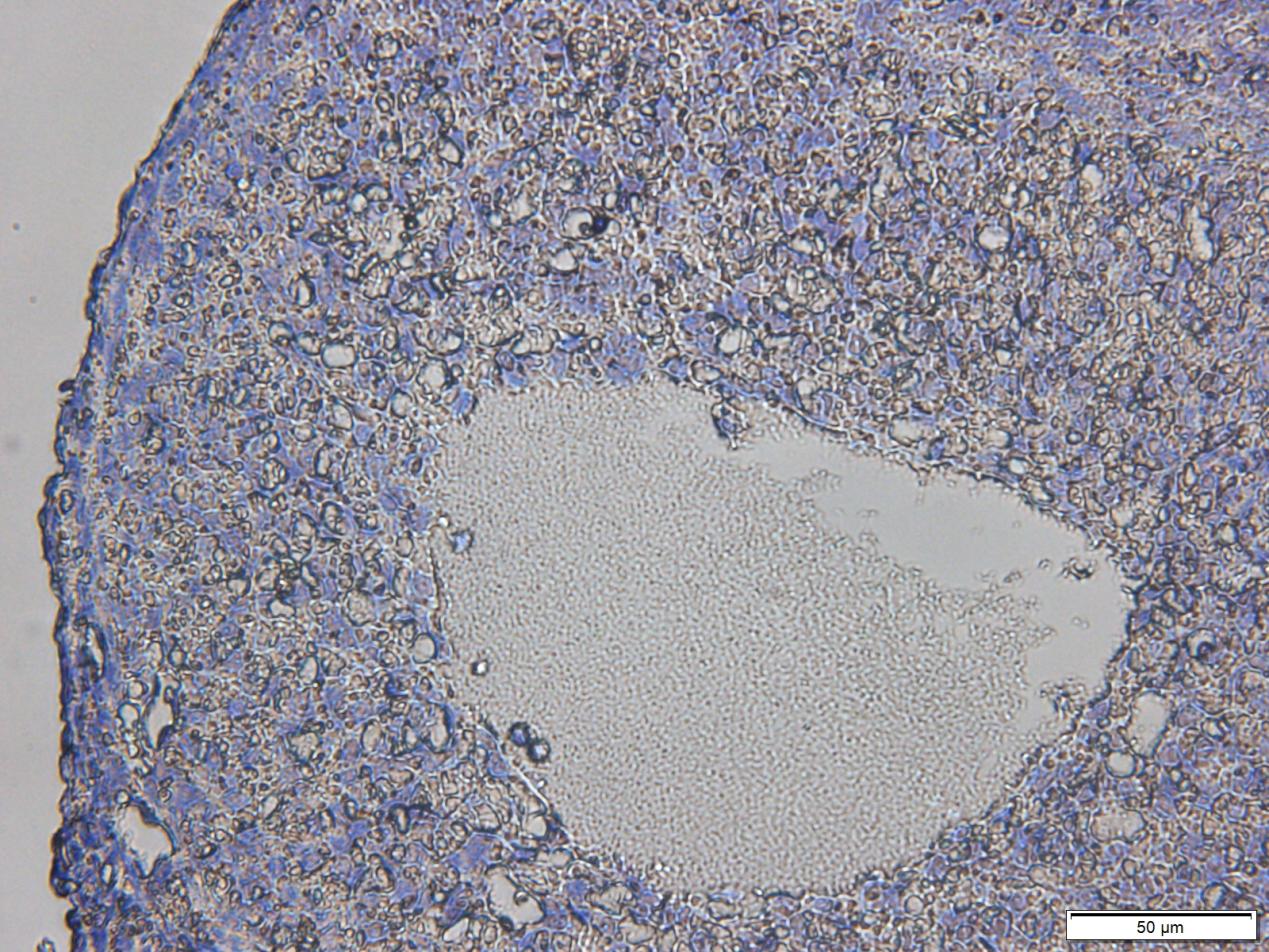


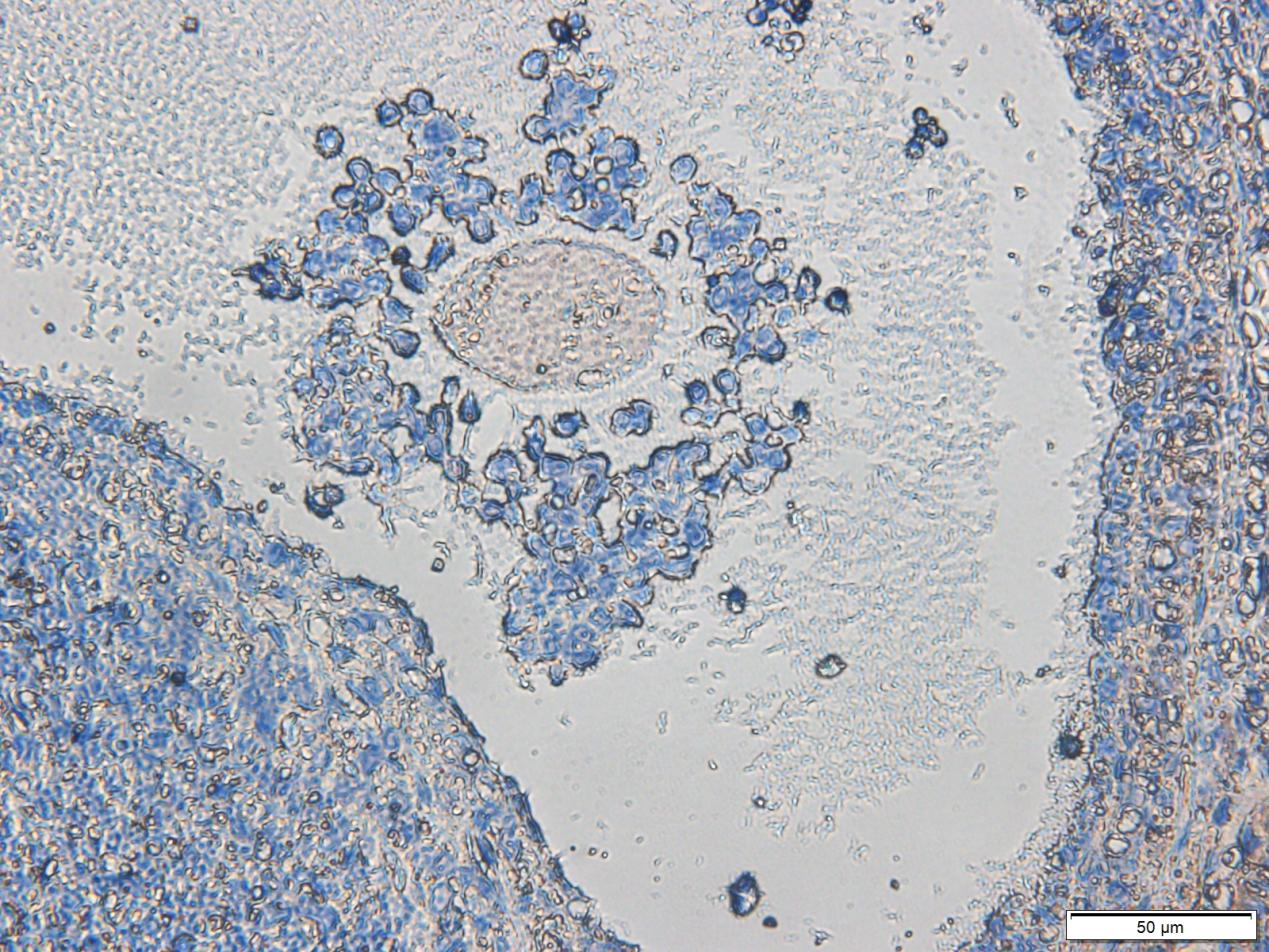


PCOS


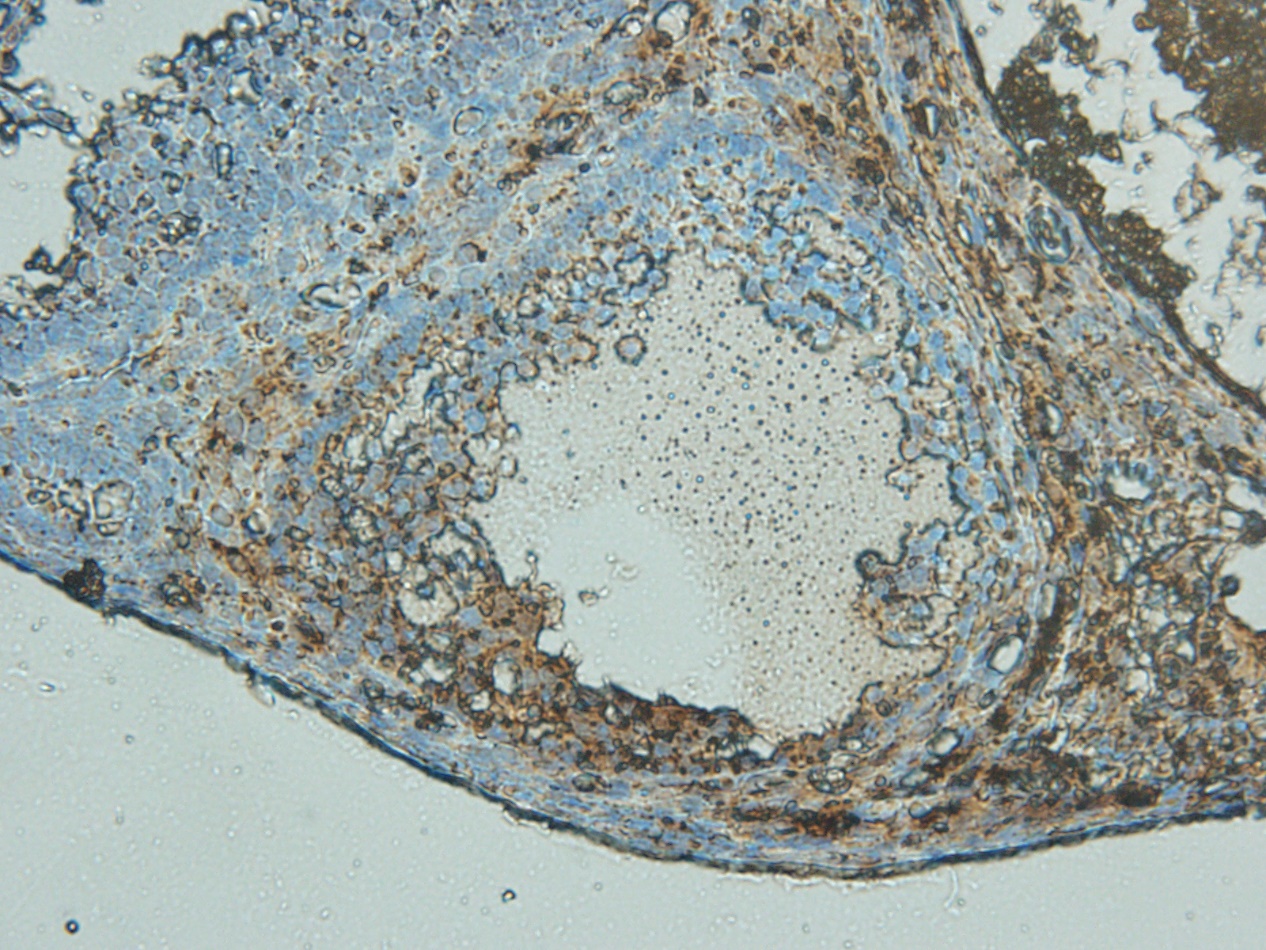

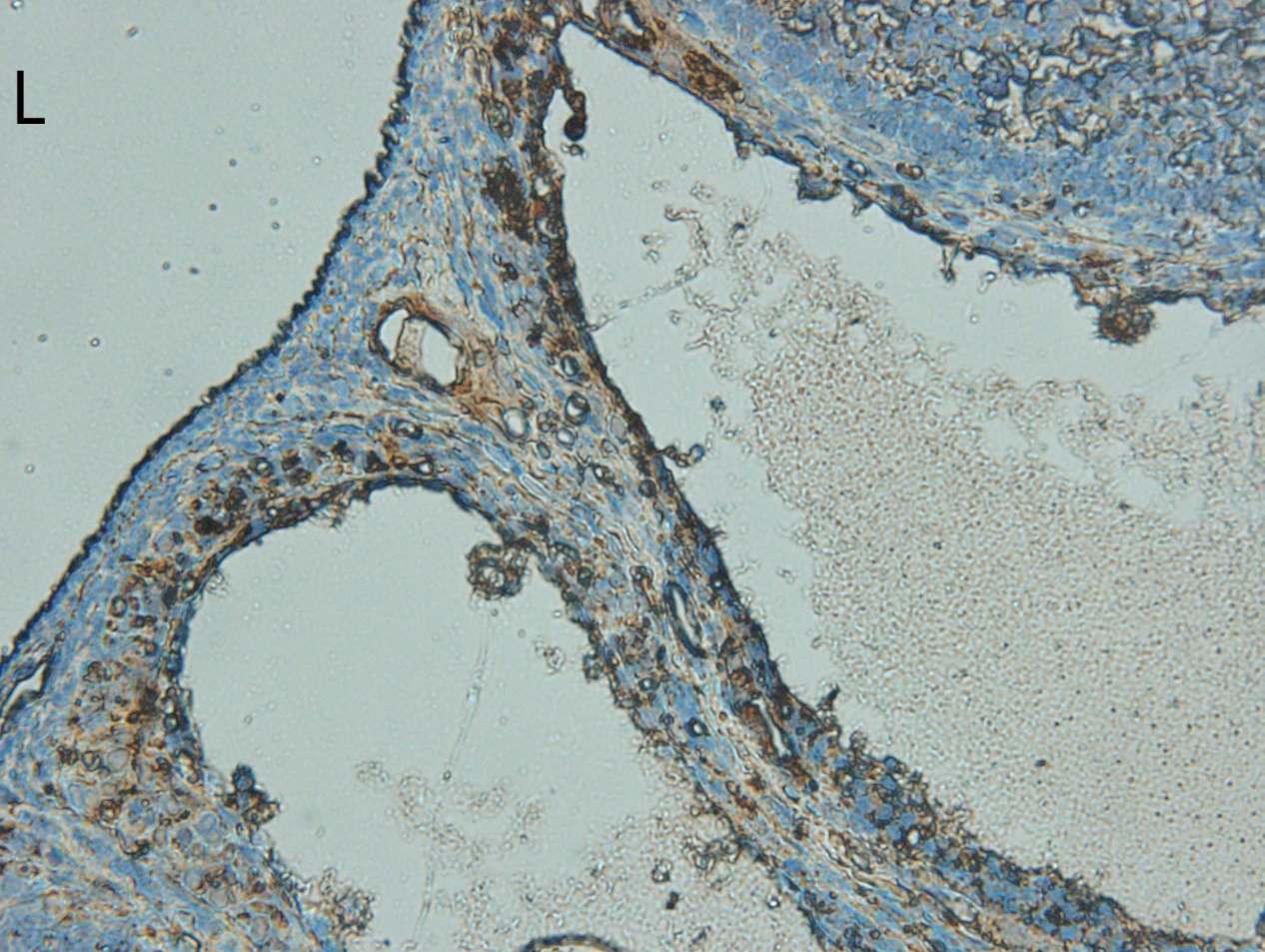


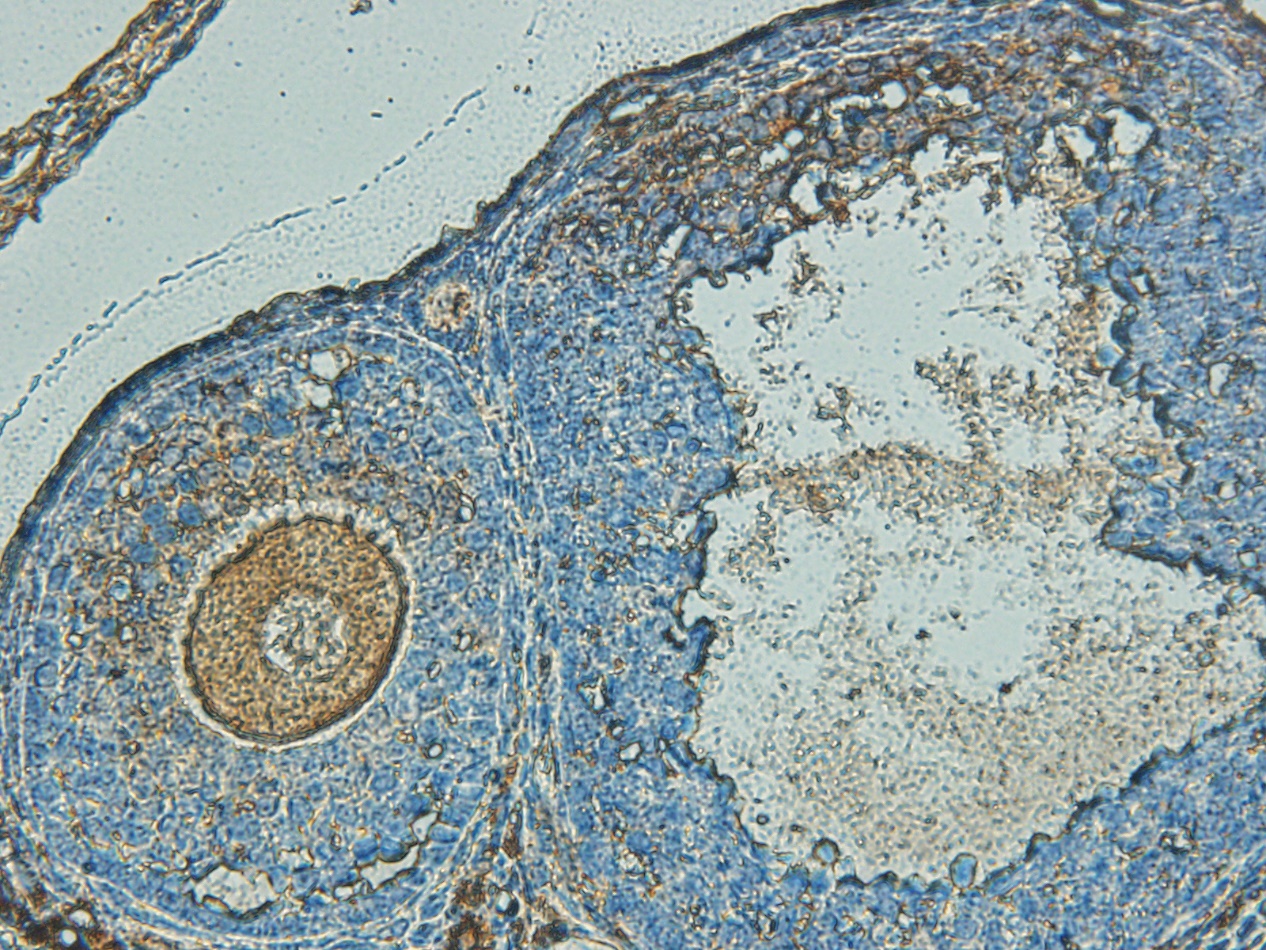


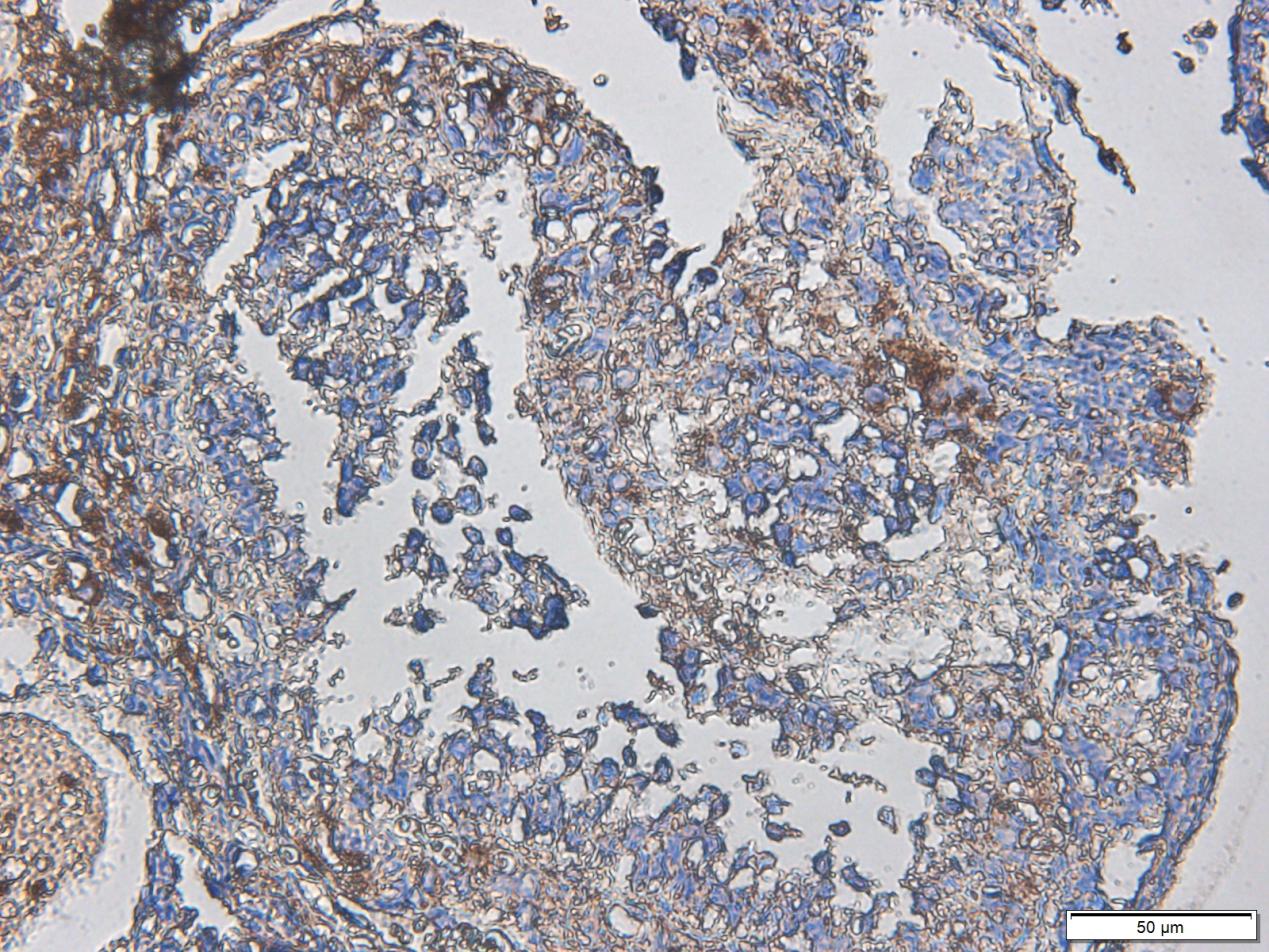


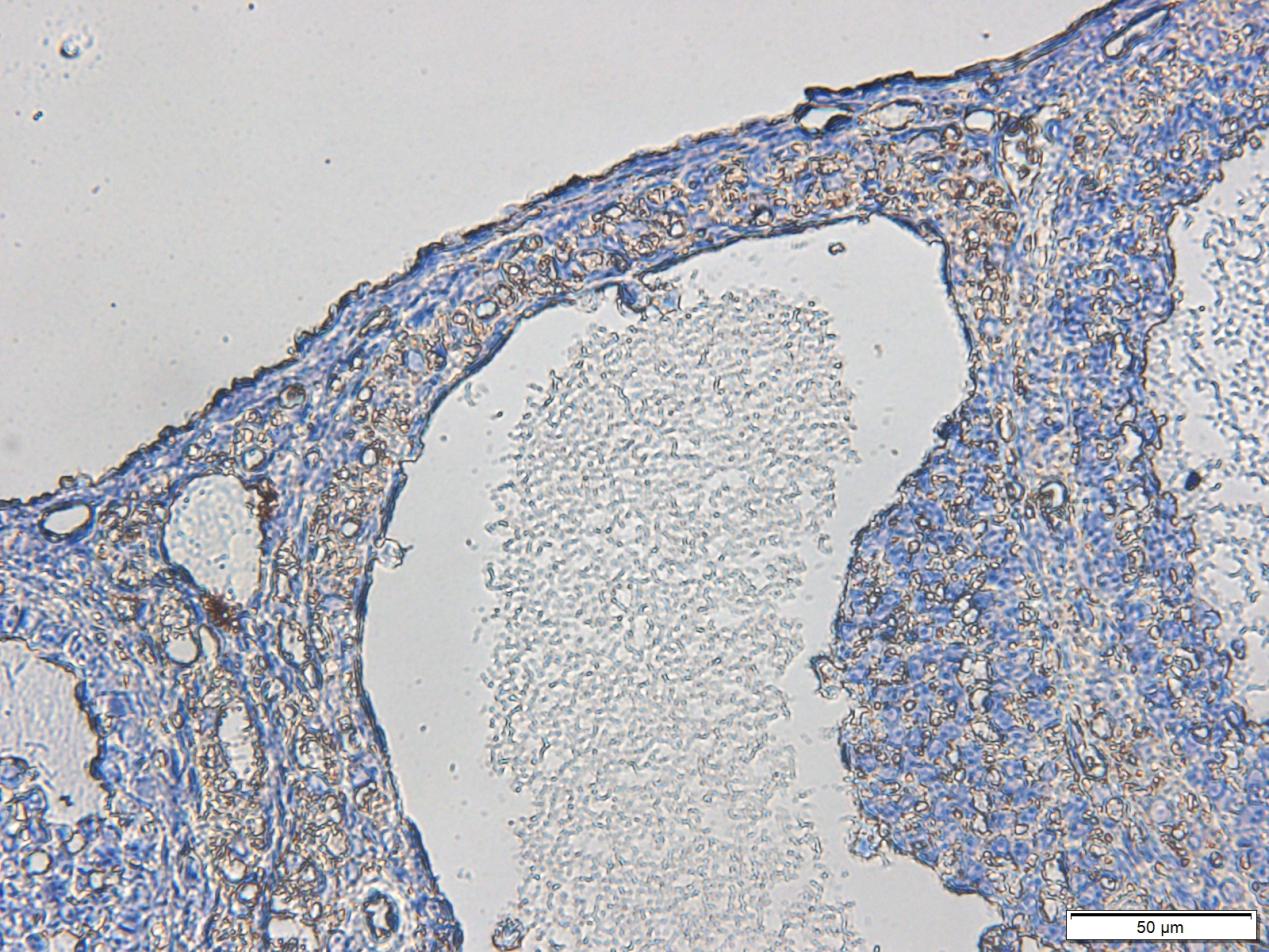

Supplement: Supplementary file 3 [file DataSheet_1.zip › 787876-data/IHC.docx]

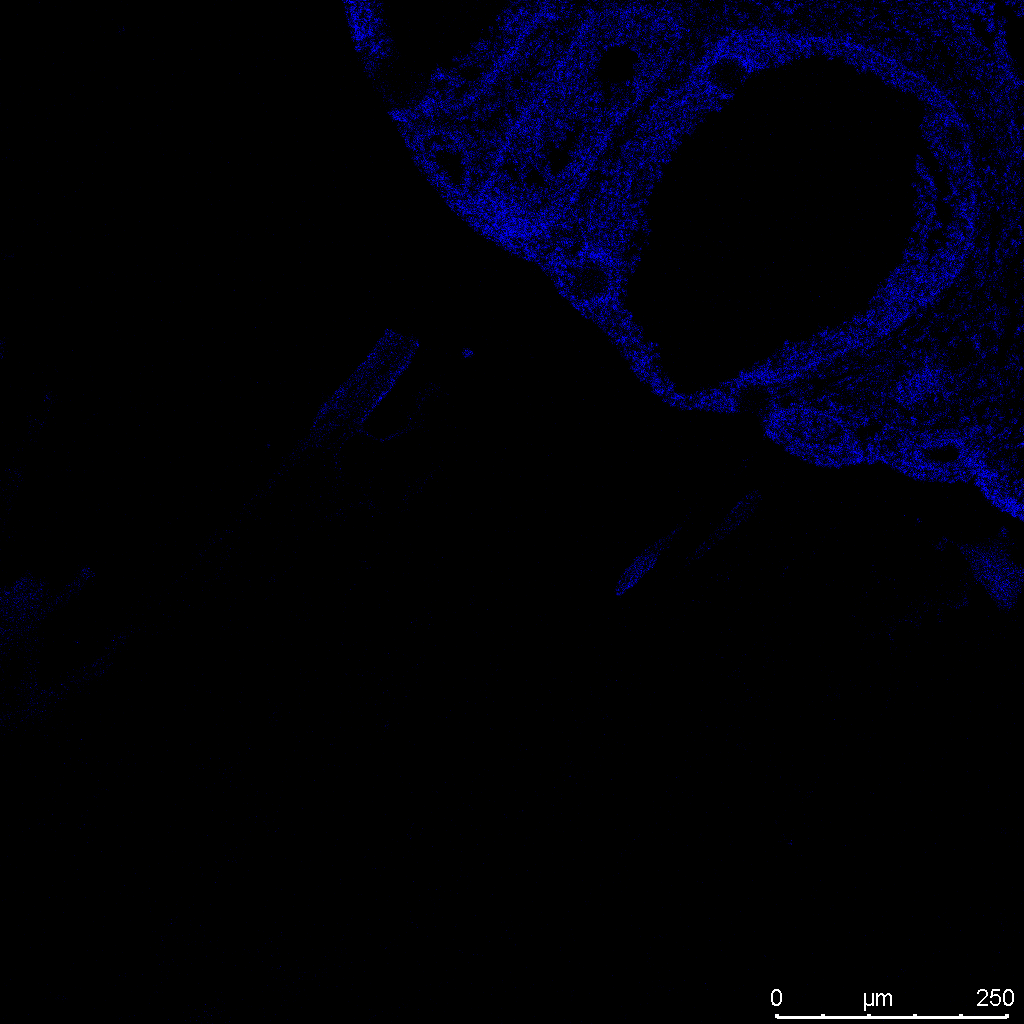

Supplement: Supplementary file 3 [file DataSheet_1.zip › 787876-data/Immunoconfocal/let 1.tif]

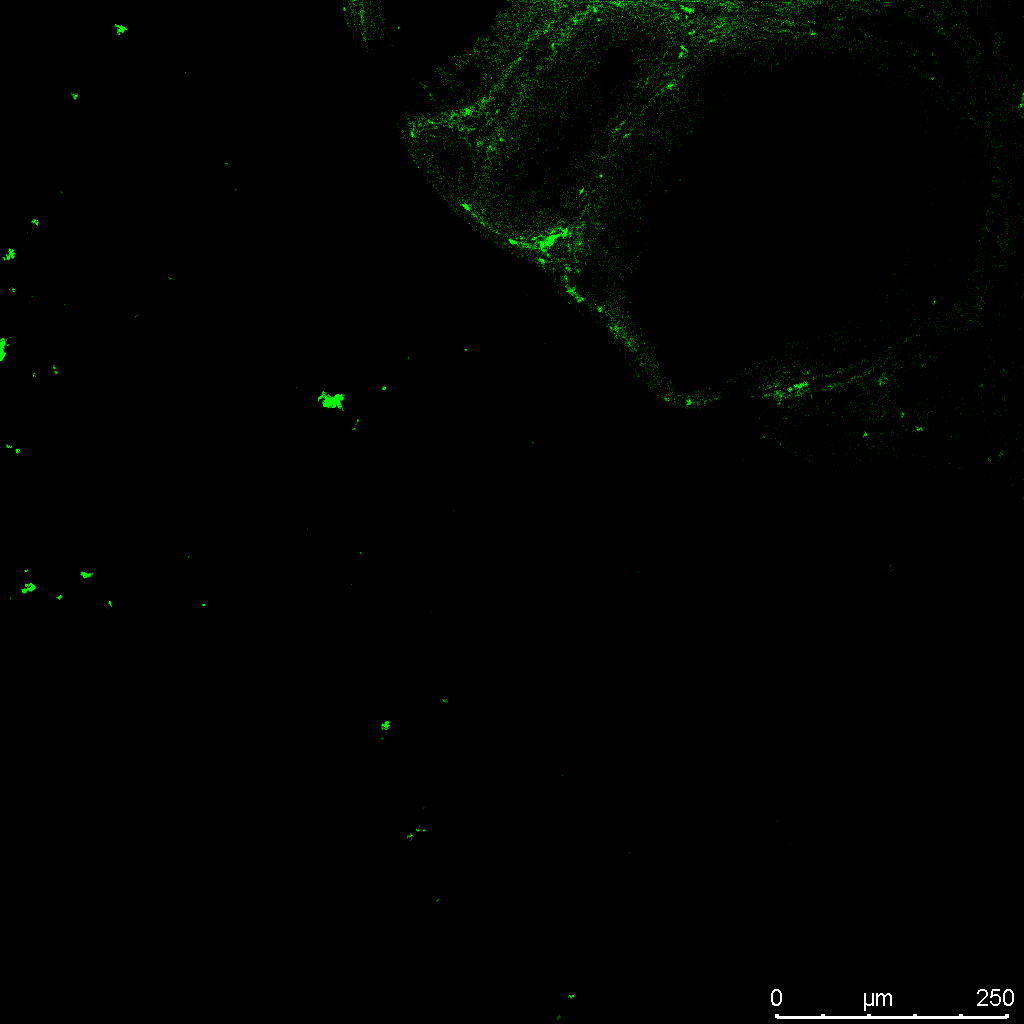

Supplement: Supplementary file 3 [file DataSheet_1.zip › 787876-data/Immunoconfocal/let2.tif]

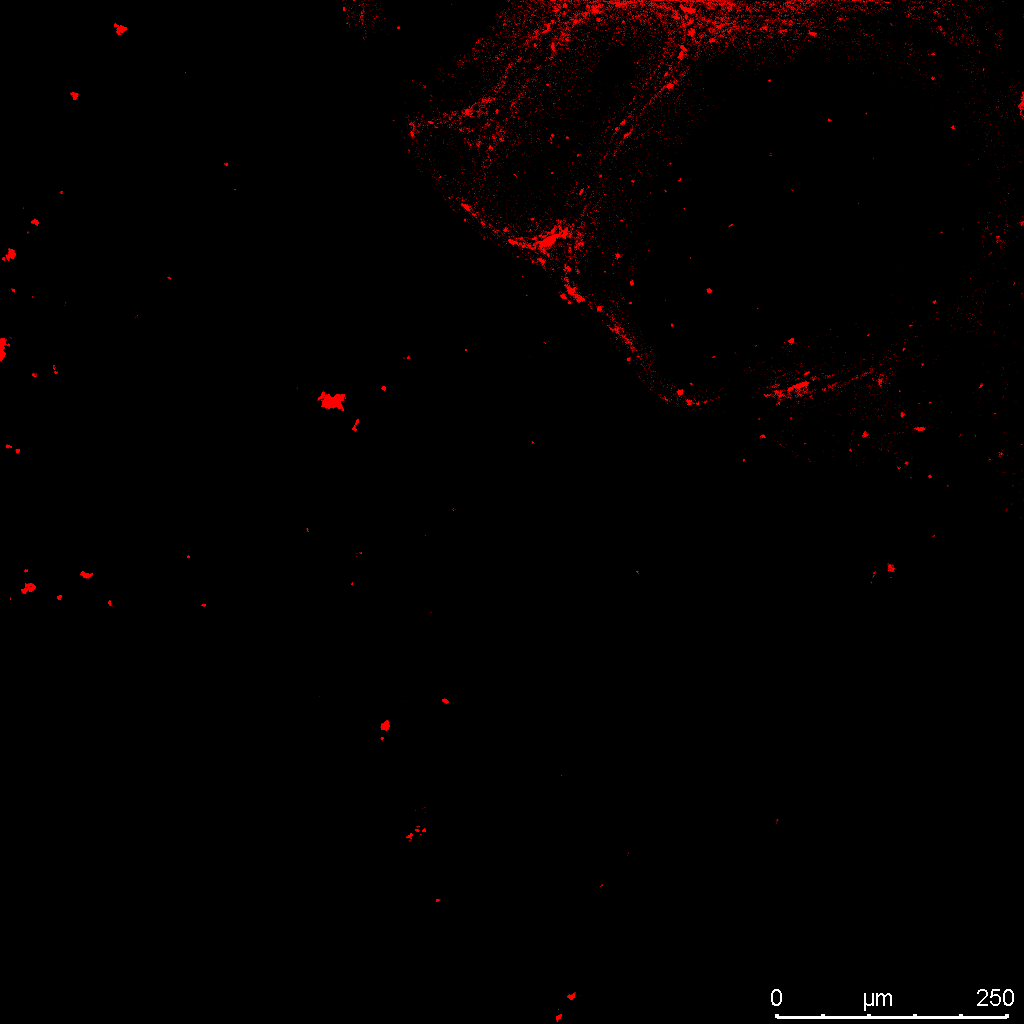

Supplement: Supplementary file 3 [file DataSheet_1.zip › 787876-data/Immunoconfocal/let3.tif]

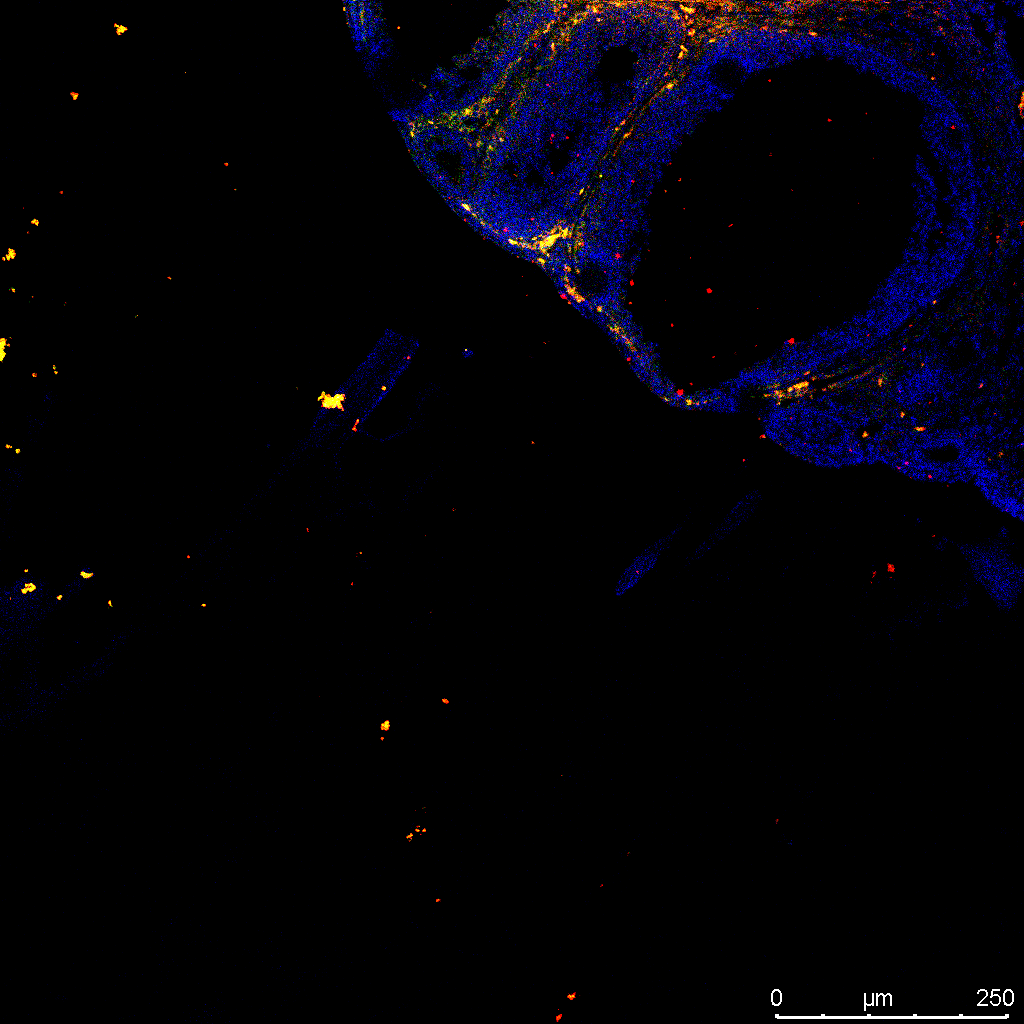

Supplement: Supplementary file 3 [file DataSheet_1.zip › 787876-data/Immunoconfocal/let4.tif]

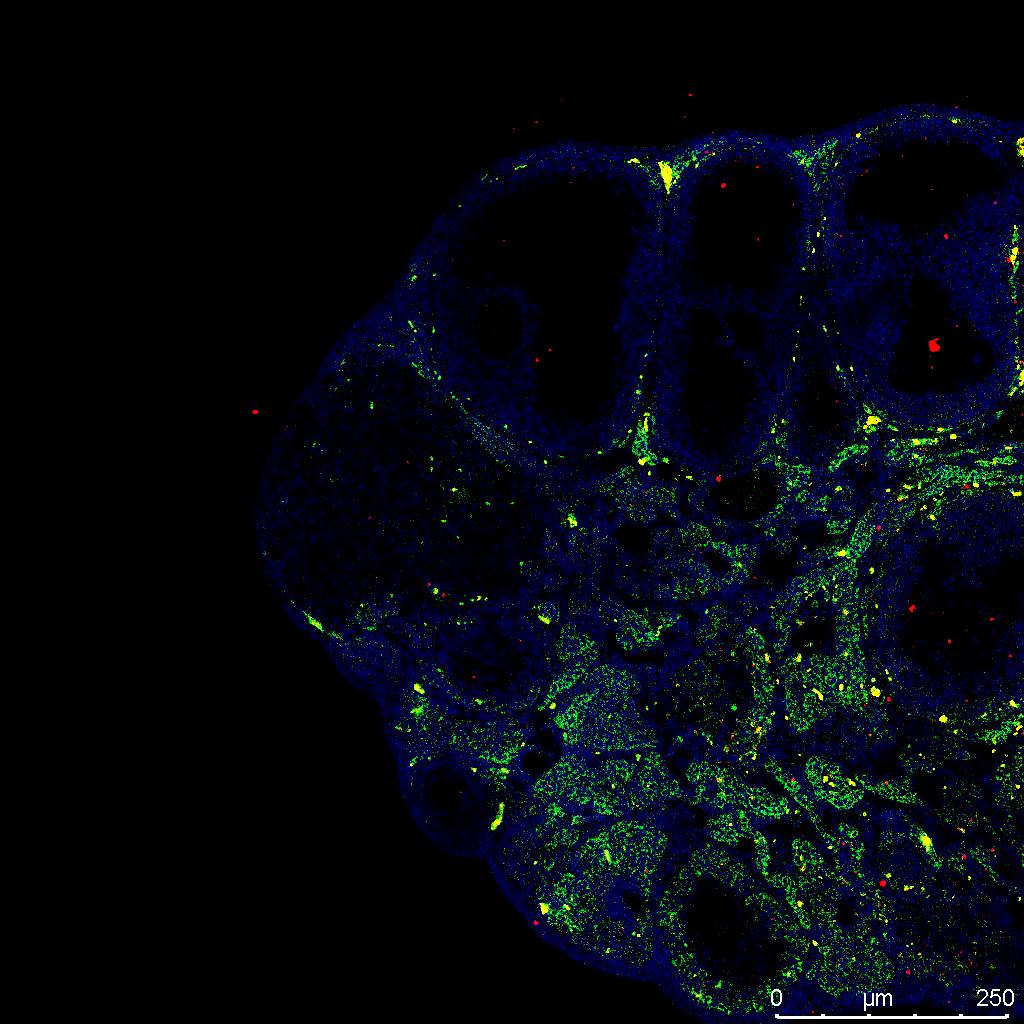

Supplement: Supplementary file 3 [file DataSheet_1.zip › 787876-data/Immunoconfocal/Project_Series004.tif]

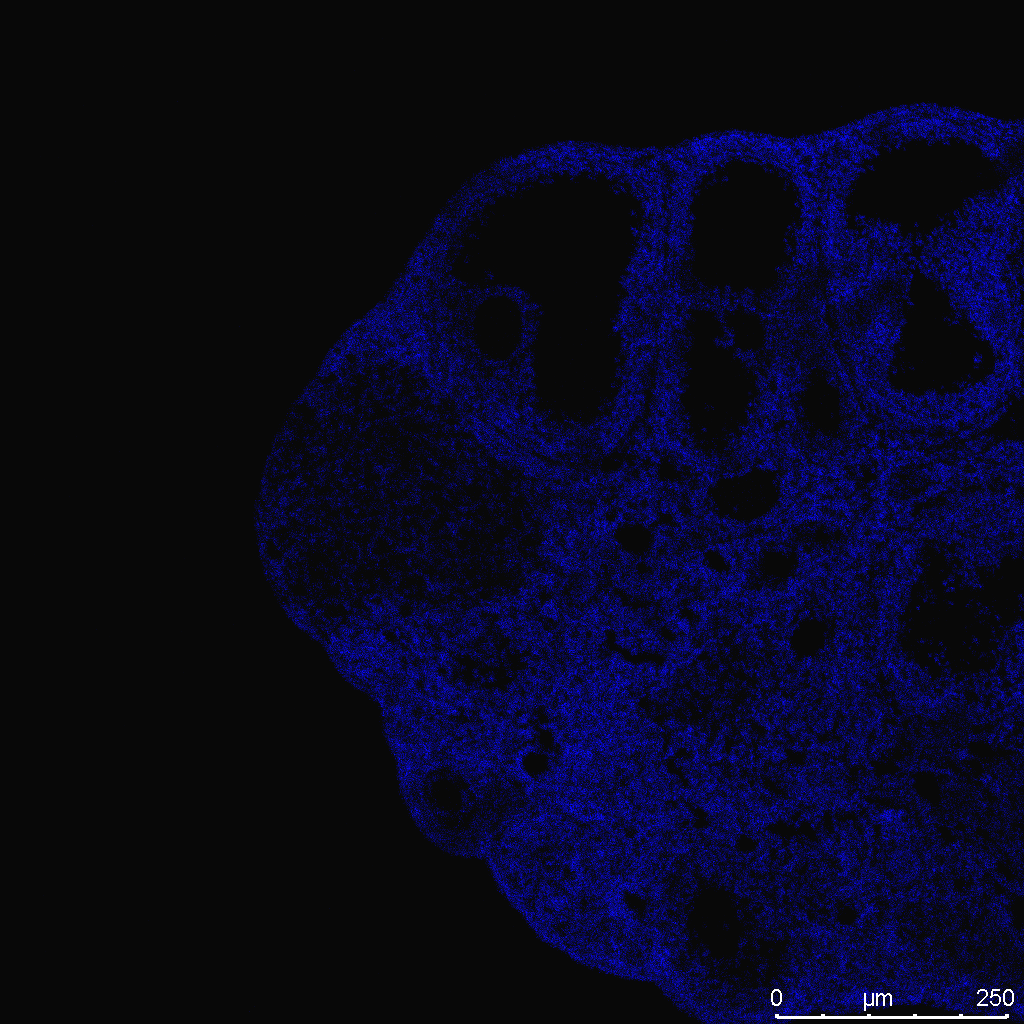

Supplement: Supplementary file 3 [file DataSheet_1.zip › 787876-data/Immunoconfocal/Project_Series004_ch00.tif]

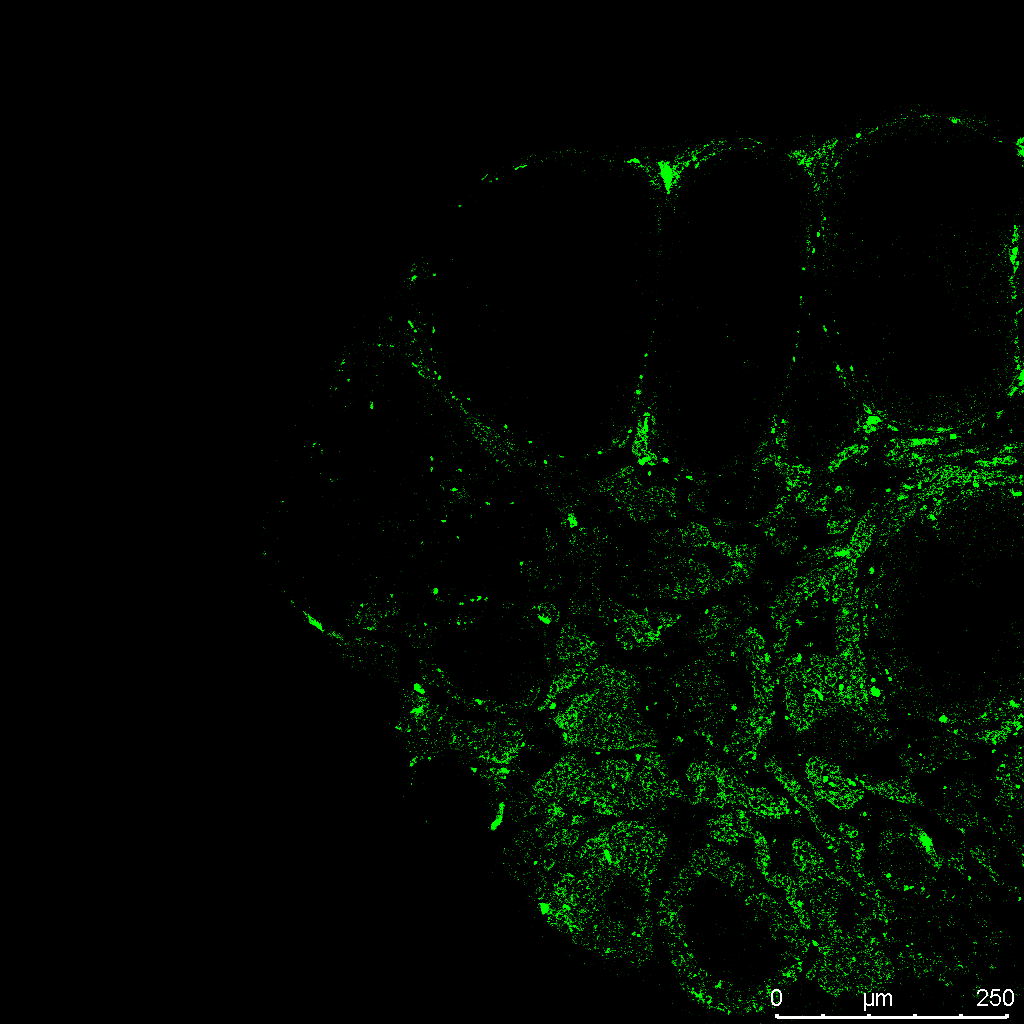

Supplement: Supplementary file 3 [file DataSheet_1.zip › 787876-data/Immunoconfocal/Project_Series004_ch01.tif]

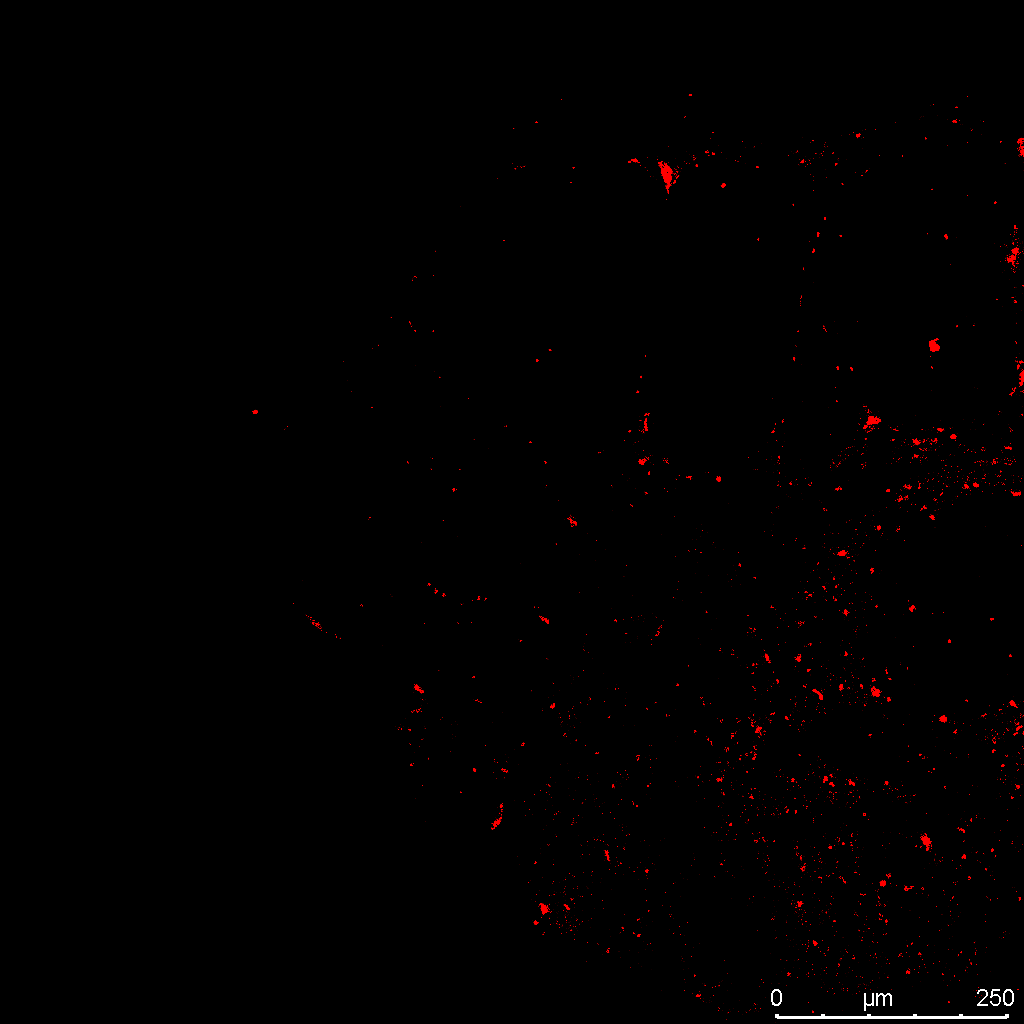

Supplement: Supplementary file 3 [file DataSheet_1.zip › 787876-data/Immunoconfocal/Project_Series004_ch02.tif]

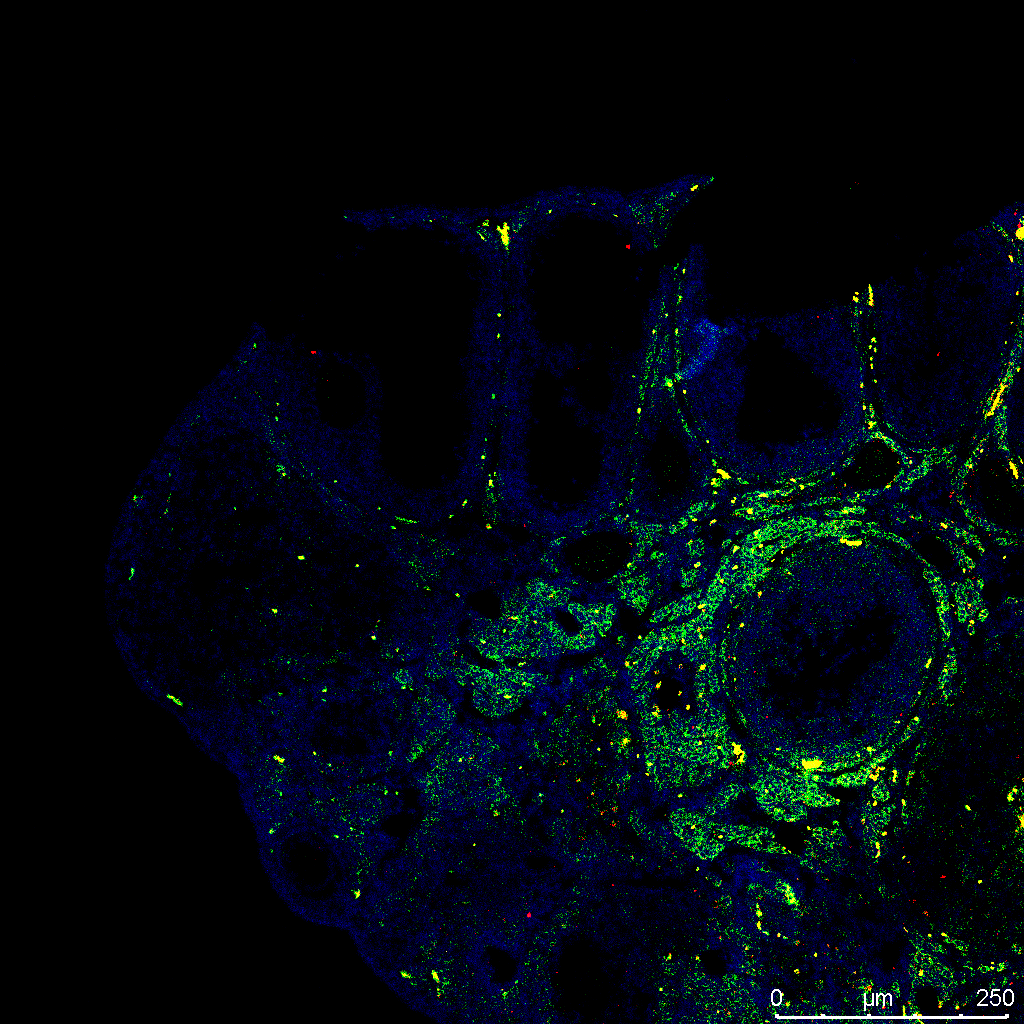

Supplement: Supplementary file 3 [file DataSheet_1.zip › 787876-data/Immunoconfocal/Project_Series005.tif]
