# Supplementary material for: IL-15 Participates in the Pathogenesis of Polycystic Ovary Syndrome by Affecting the Activity of Granulosa Cells
Source: Front Endocrinol (Lausanne). 2022 Feb 18;13:787876. doi: 10.3389/fendo.2022.787876 (PMC8894602; doi:10.3389/fendo.2022.787876)
Supplement: Supplementary Table 1 — Primer sequence list. [file Table_1.doc]

Supplementary Table1 Primer sequence list

| *Species* | *Gene* | *Sequences（5'-3'）* |
| --- | --- | --- |
| *Human* | *BAX F* | GGTGCCTCAGGATGCG |
| *Human* | *BAX R* | GGAGTCTGTGTCCACG |
| *Human* | *CYP17A1F* | GCTGCTTACCCTAGCTTATTTGT |
| *Human* | *CYP17A1R* | ACCGAATAGATGGGGCCATATTT |
| *Human* | *CYP19A1F* | TGGAAATGCTGAACCCGATAC |
| *Human* | *CYP19A1R* | AATTCCCATGCAGTAGCCAGG |
| *Human* | *FSHR F* | TCTGTCACTGCTCTAACAGGG |
| *Human* | *FSHR R* | TGCACCTTTTTGGATGACTCG |
| *Human* | *StAR F* | CCAGCTCGTGAGTAATGAATGT |
| *Human* | *StAR R* | GGGAGTGGAACCCCAATGTC |
| *Human* | *VEGF F* | GGCTGGCAACATAACAGAGAA |
| *Human* | *VEGF R* | CCCCACATCTATACACACCTCC |
| *Human* | *Ifng F* | TCGGTAACTGACTTGAATGTCCA |
| *Human* | *Ifng R* | TCGCTTCCCTGTTTTAGCTGC |
| *Human* | *Tnfa F* | CCTCTCTCTAATCAGCCCTCTG |
| *Human* | *Tnfa R* | GAGGACCTGGGAGTAGATGAG |
| *Human* | *IL-1bF* | ATGATGGCTTATTACAGTGGCAA |
| *Human* | *IL-1bR* | GTCGGAGATTCGTAGCTGGA |
| *Human* | *IL-6 F* | ACTCACCTCTTCAGAACGAATTG |
| *Human* | *IL-6 R* | CCATCTTTGGAAGGTTCAGGTTG |
| *Human* | *IL-18 F* | TCTTCATTGACCAAGGAAATCGG |
| *Human* | *IL-18 R* | TCCGGGGTGCATTATCTCTAC |
| *Human* | *TLR4 F* | AGACCTGTCCCTGAACCCTAT |
| *Human* | *TLR4 R* | CGATGGACTTCTAAACCAGCCA |
| *Human* | *IL-15 F* | TTGGGAACCATAGATTTGTGCAG |
| *Human* | *IL-15 R* | GGGTGAACATCACTTTCCGTAT |
| *Mouse* | *BAX F* | AGACAGGGGCCTTTTTGCTAC |
| *Mouse* | *BAX R* | AATTCGCCGGAGACACTCG |
| *Mouse* | *CYP17A1F* | AGTGCTCGTGAAGAAGGGGA |
| *Mouse* | *CYP17A1R* | TTTCCTTGGTCCGACAAGAGG |
| *Mouse* | *CYP19A1F* | ACTACATCTCCCGATTCGGCA |
| *Mouse* | *CYP19A1R* | GGGTCAACACATCCACGTAGC |
| *Mouse* | *FSHR F* | TGCTCTAACAGGGTCTTCCTC |
| *Mouse* | *FSHR R* | TCTCAGTTCAATGGCGTTCCG |
| *Mouse* | *StAR F* | CGTGAGCGTGCGCTGTACCA |
| *Mouse* | *StAR R* | TGACACCACTCTGCTCCGGCA |
| *Mouse* | *VEGF F* | GTGAGGTGTGTATAGATGTGGGG |
| *Mouse* | *VEGF R* | ACGTCTTGCTGAGGTAACCTG |
| *Mouse* | *Ifng F* | TCCTCGCCAGACTCGTTTTC |
| *Mouse* | *Ifng R* | ACGGCTCCCAAGTTAGAATCT |
| *Mouse* | *Tnfa F* | AGGGTCTCCGCCATAGAACT |
| *Mouse* | *Tnfa R* | CCACCACGCTCTTCTGTCTAC |
| *Mouse* | *IL-1bF* | TTCAGGCAGGCAGTATCACTC |
| *Mouse* | *IL-1bR* | GAAGGTCCACGGGAAAGACAC |
| *Mouse* | *IL-6 F* | GCTACCAAACTGGATATAATCAGGA |
| *Mouse* | *IL-6 R* | CCAGGTAGCTATGGTACTCCAGAA |
| *Mouse* | *IL-18 F* | GACTCTTGCAACTTCAAGG |
| *Mouse* | *IL-18 R* | CAGGCTGTCTTTTCTCAACGA |
| *Mouse* | *TLR4 F* | TTTGTCCCACAATGAGCTAAAGG |
| *Mouse* | *TLR4 R* | TTCTTTGCATATAGGCAGGGC |
| *Mouse* | *IL-15 F* | ACATCCATCTCGTGCTACTTGT |
| *Mouse* | *IL-15 R* | GCCTCTGTTTTAGGGAGACCT |
